# Supplementary material for: High prevalence of focal and multi-focal somatic genetic variants in the human brain
Source: Nat Commun. 2018 Oct 15;9:4257. doi: 10.1038/s41467-018-06331-w (PMC6189186; doi:10.1038/s41467-018-06331-w)
Supplement: Supplementary file 1 — Supplementary Information [file 41467_2018_6331_MOESM1_ESM.pdf]

Supplementary Tables

Supplementary Table 1. The 102 Neurodegenerative disease and cancer genes sequenced. Both sets were included on the Accuracy and Content Enhanced (ACE) and Haloplex^{HS} platforms. Left = neurodegenerative disease genes. Right = cancer genes. Key – AD: Alzheimer's disease. DLB: Dementia with Lewy Bodies. FTD-ALS: Frontotemporal dementia – Amyotrophic lateral sclerosis. PD: Parkinson's disease. CJD – Creutzfeldt Jakob Disease.

Case Gene	Disorder	Control Gene	Associated disorder	Associated with haematological malignancy
ANG	FTD-ALS	*ALK*	Lymphoma	Y [1]
APOE	AD / DLB	*AR*	Prostate cancer /	N
APP	AD	*CCND1*	B-cell leukaemia	Y [2]
DAO	FTD-ALS	*BCL2*	B-cell leukaemia	Y[2]
DCTN1	FTD-ALS	*BRCA1*	Breast cancer	N
EIF4G1	PD	*BRCA2*	Breast cancer	N
EWSR1	FTD-ALS	*CCNE1*	Breast cancer	N
FUS	FTD-ALS	*CDH1*	Stomach cancer	N
GBA	PD/DLB	*CDK6*	Leukaemia	Y [3]
GRN	FTD-ALS	*CDKN2A*	Pancreatic cancer	N
HNRNPA1	FTD-ALS	*CDKN2B*	Myelodysplastic syndrome	Y [4]
HNRNPA2B1	FTD-ALS	*DNMT3A*	Acute Myeloid Leukaemia /	Y [5]
PON3	FTD-ALS	*ERBB2*	Lung cancer	N
SQSTM1	FTD-ALS	*ERG*	Acute myeloid leukaemia	Y [6]
NOTCH3	CADASIL	*ESR1*	Breast cancer	N
PINK1	PD	*ETV4*	Sarcoma	N
UBQLN2	FTD-ALS	*ETV6*	Acute myeloid leukaemia	Y [7]
GIGYF2	PD	*EZH2*	Myelodysplastic syndrome	Y [8]
DPP6	FTD-ALS	*JAK2*	Acute myeloid leukaemia	Y [9]
C9orf72	FTD-ALS	*JAK3*	Severe Combined	Y [10]
MAPT	FTD-ALS	*KDR*	Haemangioma	N
PARK2	PD	*MEN1*	Multiple Endocrine	N
PFN1	FTD-ALS	*MITF*	Melanoma	N
PON1	FTD-ALS	*MPL*	Myeloproliferation	Y [11]
PON2	FTD-ALS	*MYC*	Burkitt Lymphoma	Y [12]
PRNP	CJD	*DDR2*	No known cancer	N
PRPH	FTD-ALS	*PDGFRB*	Myofibromatosis / IBGC	N
PSEN1	AD	*RARA*	Acute myeloid leukaemia	Y [13]
PSEN2	AD	*RET*	Multiple Endocrine	N
SMN1	FTD-ALS	*ROS1*	Myocardial infarction	N
SNCA	PD	*SMO*	Basal cell carcinoma	N
SOD1	FTD-ALS	*AURKA*	Colon cancer	N
SORL1	AD	*SYK*	No known cancer	N
SPR	PD	*NKX2-1*	Medullary cancer	N
UCHL1	PD	*TMPRSS2*	Thyroid cancer	N
VCP	FTD-ALS	*TP53*	Age related clonal	Y [14]
TAF15	FTD-ALS	*TET2*	Myelodysplastic syndrome	Y [5]
UNC5C	AD	*NUTM1*	Midline tumours	N
VAPB	FTD-ALS	*KMT2A*	Acute myeloid leukaemia	Y [15]
MATR3	FTD-ALS	*CEBPA*	Acute myeloid leukaemia	Y [16]
FIG4	FTD-ALS	*NOTCH2*	Alagille Syndrome	N
OPTN	FTD-ALS	*RUNX1*	Acute myeloid leukaemia	Y [17]
SIGMAR1	FTD-ALS	*NOTCH1*	Adams-Oliver syndrome	N
PARK7	PD	*WT1*	Wilms tumour	N
SETX	FTD-ALS	*ARAF*	No known cancer	N
TARDBP	FTD-ALS	*EPHA3*	No known cancer	N
FBXO7	PD			
CHMP2B	FTD-ALS			
COQ2	FTD-ALS			
HTRA2	PD			
TREM2	PD/DLB			
VPS35	PD			
ALS2	FTD-ALS			
SPG11	PD			
CHCHD10	FTD-ALS			
LRRK2	PD			

Supplementary Table 2. Sensitivity and specificity for each calling algorithm. Determined using HapMap CEPH cell lines NA12877 & NA12878.

VAF	Caller	Sensitivity	Specificity
0.2%	Mutect2	0.3158	0.9999
0.5%	Mutect2	0.8070	1.0000
1.0%	Mutect2	0.9737	1.0000
2.0%	Mutect2	0.9825	1.0000
5.0%	Mutect2	1.0000	1.0000
0.2%	Mutect2 and Varscan (concordant call)	0.2544	1.0000
0.5%	Mutect2 and Varscan (concordant call)	0.7807	1.0000
1.0%	Mutect2 and Varscan (concordant call)	0.9386	1.0000
2.0%	Mutect2 and Varscan (concordant call)	0.9474	1.0000
5.0%	Mutect2 and Varscan (concordant call)	1.0000	1.0000
0.2%	Mutect2 or Varscan	0.5614	0.9998
0.5%	Mutect2 or Varscan	0.9298	1.0000
1.0%	Mutect2 or Varscan	0.9825	1.0000
2.0%	Mutect2 or Varscan	1.0000	1.0000
5.0%	Mutect2 or Varscan	1.0000	1.0000
0.2%	Varscan	0.5000	0.9998
0.5%	Varscan	0.9035	1.0000
1.0%	Varscan	0.9474	1.0000
2.0%	Varscan	0.9649	1.0000
5.0%	Varscan	1.0000	1.0000
0.2%	deepSNV	0.1579	1.0000
0.5%	deepSNV	0.5965	1.0000
1.0%	deepSNV	0.7368	1.0000
2.0%	deepSNV	0.9298	1.0000
5.0%	deepSNV	1.0000	1.0000

Supplementary Table 3. Clinical and neuropathological data for all cases and controls. Both *ante mortem* and *post mortem* diagnoses are given, together with the Braak tau [18] , Thal phase [19], CERAD score [20], Braak Lewy body stage [21], and the McKeith stage [22] for each brain. Key – AD: Alzheimer's disease, PD: Parkinson's disease, PDD: Parkinson's disease dementia, DLB: Dementia with Lewy Bodies, MCI: Mild Cognitive Impairment.

**Case**	**Clinical diagnosis**	**Neuro-path diagnosis**	**Age at death (years)**	**Braak stage (tau)**	**Thal Phase**	**CERAD**	**Braak Lewy Body Stage**	**McKeith Stage**
1	Control	Control	65	1	0	negative	0	negative
2	Control	Control	88	2	3	negative	0	negative
3	Control	Control	64	1	0	negative	0	negative
4	Control	Control	88	2	1	negative	0	negative
5	Control	Control	78	0	1	negative	0	negative
6	Control	Control	89	3	2	negative	0	negative
7	Control	Control	97	2	2	negative	0	negative
8	Control	Control	85	1	2	negative	0	negative
9	Control	Control	73	0	0	negative	0	negative
10	Control	Control	80	3	2	negative	0	negative
11	Control	Control	81	1	0	negative	0	negative
12	Control	Control	81	2	0	negative	0	negative
13	Control	Control	80	1	1	negative	0	negative
14	Control	Control	93	3	1	negative	0	negative
15	AD	AD	68	6	5	frequent	0	negative
16	AD	AD	96	6	5	frequent	0	negative
17	AD	AD	91	6	5	frequent	0	negative
18	AD	AD	83	6	5	frequent	0	negative
19	AD	AD	78	6	5	frequent	0	negative
20	AD	AD	90	6	5	frequent	0	negative
21	AD	AD	89	6	5	frequent	0	negative
22	AD	AD	86	6	5	frequent	0	negative
23	AD	AD	92	6	5	frequent	0	negative
24	AD	AD	92	6	5	frequent	0	negative
25	AD	AD	81	6	5	frequent	0	negative
26	AD	AD	75	6	5	frequent	0	negative
27	AD	AD	84	6	5	frequent	0	negative
28	AD	AD	85	6	5	frequent	0	negative
29	AD	AD	93	6	5	frequent	0	negative
30	AD	AD	86	6	5	frequent	0	negative
31	AD	AD	84	6	5	frequent	0	negative
32	AD	AD	83	6	4	frequent	0	negative
33	AD	AD	77	6	5	frequent	0	negative
34	AD	AD	85	6	4	frequent	0	negative
35	PD	PD	90	2	0	negative	4	limbic
36	PD+MCI	DLB	80	3	0	negative	6	neocortical
37	DLB	DLB	92	1	0	negative	5	limbic
38	PDD	DLB	86	1	3	negative	5	neocortical
39	PD	PD	70	2	0	negative	4	limbic
40	DLB	DLB	76	2	3	negative	4	limbic
41	DLB	DLB	78	3	4	moderate	6	neocortical
42	DLB	DLB+AD	79	6	5	frequent	6	neocortical
43	PDD	DLB+AD	77	6	5	frequent	6	neocortical
44	DLB	DLB+AD	78	6	5	frequent	4	limbic
45	DLB	DLB+AD	78	6	5	frequent	4	neocortical
46	DLB	DLB+AD	67	6	5	frequent	6	neocortical
47	DLB	DLB	81	3	4	moderate	6	neocortical
48	DLB	DLB	81	3	3	moderate	6	neocortical
49	PDD	DLB	76	3	1	negative	5	neocortical
50	PDD	DLB	83	4	5	moderate	6	neocortical
51	DLB	DLB	73	3	1	negative	6	neocortical
52	DLB	DLB+AD	78	5	4	frequent	6	neocortical
53	DLB	DLB	91	3	4	moderate	6	neocortical
54	PD	PD	83	3	3	negative	4	limbic
**Cont: Mean (SD)**			81.6 (9.58)	1.6 (1.0)	1.1 (1.0)		0.00 (0.00)	
**AD: Mean (SD)**			84.9 (6.8)	**6.0 (0.0) ****	**4.9 (0.3)****		0.00 (0.00)	
**LB: Mean (SD)**			79.9 (6.5)	**3.6 (1.7) ****	**3.00 (1.9)***		**5.25 (0.9)****	

Supplementary Table 4. Sample sizes following quality control. In total 173 brain samples and 6 paired blood samples from 54 individuals remained (Controls: n=14, Alzheimer's disease: n=20, Lewy body disease: n=20).

	Cerebellum	Entorhinal cortex	Frontal cortex	Medulla	Cingulate	Blood	Total within each disease cohort
**Controls**	14	14	14	7	5	2	56
**Alzheimer disease**	20	19	18	0	0	1	58
**Lewy body disease**	20	20	0	17	5	3	65
**Total**	54	53	32	24	10	6	179

Supplementary Table 5. Variants detected within the study. Gene and base position (in GRCh37 build) are described, together with the relative minor allele frequency within the ExAC and ESP6500 server. Four *in-silico* prediction tools for pathogenicity are also shown (SIFT [23], LRT [24], MutPred [25] and CADD [26]) are also shown. Right: relevant clinical data for the specified variant. Both case number relating to the specific case (Supplementary Table 1), the mutation class (Single Region Mutations, SRM; or Multiple Region Mutations, MRM), and whether the variant was sequenced at not present (red) or sequenced and present (blue). Grey boxes signify that the region was not sequenced.

Variant data																Case data								
Case /Control Gene	Gene	Chr	Base Position	Ref allele	Alt allel	Mutation	ExAC MAF	ESP6500 MAF	SIFT_Score	SIFT_Pred	LRT_Score	LRT_Pred	MUT_Score	Mut_Pred	CADD_phred	Case number	Cohort	Mutation class	Cerebellum	Entorhinal Cortex	Frontal Cortex	Medulla	Cingulate gyrus	Blood
Case	*EIF4G1*	3	184052604	G	A	NS	.	.	0.1	T	0	D	1	D	21.8	28	AD	SRM						
Case	*LRRK2*	12	40709048	G	A	S	.	.	.	.	.	.	.	.	.	24	AD	SRM						
Case	*NOTCH3*	19	15276289	C	T	NS	0.00000830	.	0	D	0.007	N	1	D	28.2	45	LB	SRM						
Case	*SETX*	9	135206659	C	A	I	.	.	.	.	.	.	.	.	.	34	AD	SRM						
Case	*SORL1*	11	121421298	C	T	NS	0.00000827	.	0.03	D	0	D	1	D	22	9	Cont	SRM						
Case	*UCHL1*	4	41262768	C	T	S	0.00002471	0.00008	.	.	.	.	.	.	.	17	AD	SRM						
Case	*VPS35*	16	46705662	G	A	S	.	.	.	.	.	.	.	.	.	18	AD	SRM						
Case	*TAF15*	17	34171702	A	C	S	0.00001069	.	.	.	.	.	.	.	.	12	Cont	MRM						
Control	*ARAF*	X	47424757	T	A	UTR	.	.	.	.	.	.	.	.	.	51	LB	SRM						
Control	*BRCA1*	17	41234513	C	T	NS	0.00000824	.	0.05	D	0.015	N	1	D	18.54	41	LB	SRM						
Control	*BRCA2*	13	32950860	C	T	NS	0.00004970	0.00008	0.22	T	0.513	N	1	N	11.47	46	LB	SRM						
Control	*DNMT3A*	2	25464529	C	T	NS	0.00000839	.	0	D	0	D	1	D	36	49	LB	SRM						
Control	*ERBB2*	17	37882110	C	T	I	0.00010000	.	.	.	.	.	.	.	.	34	AD	SRM						
Control	*PDGFRB*	5	149497364	A	ATGGC	FSI	.	.	.	.	.	.	.	.	.	17	AD	SRM						
Control	*RET*	10	43597857	C	T	S	0.00020000	0.00050	.	.	.	.	.	.	.	7	Cont	SRM						
Control	*ROS1*	6	117724357	C	T	S	0.00007415	.	.	.	.	.	.	.	.	34	AD	SRM						
Control	*ROS1*	6	117678995	T	C	NS	.	.	0.71	T	0.36	N	1	D	6.403	35	LB	SRM						
Control	*SYK*	9	93607773	G	A	NS	0.00000824	.	0.18	T	0	D	1	D	28.5	53	LB	SRM						
Control	*TET2*	4	106158207	C	T	S	0.00003301	.	.	.	.	.	.	.	.	18	AD	SRM						

Variant data																Case data								
Case /Control Gene	Gene	Chr	Base Position	Ref allele	Alt allel	Mutation	ExAC MAF	ESP6500 MAF	SIFT_Score	SIFT_Pred	LRT_Score	LRT_Pred	MUT_Score	Mut_Pred	CADD_phred	Case number	Cohort	Mutation class	Cerebellum	Entorhinal Cortex	Frontal Cortex	Medulla	Cingulate gyrus	Blood
Control	*TET2*	4	106155806	AT	A	FSD	.	.	.	.	.	.	.	.	.	8	Cont	SRM						
Control	*TET2*	4	106190900	C	T	NS	.	.	0	D	.	.	1	D	28	8	Cont	SRM						
Control	*TP53*	17	7578281	G	T	NS	.	.	0	D	0	D	1	D	15.26	38	LB	SRM						
Control	*TP53*	17	7578410	T	A	NS	.	.	0	D	0.006	N	0.969	D	19.94	54	LB	SRM						
Control	*DNMT3A*	2	25461999	C	G	NS	.	.	.	.	0	D	1	D	33	29	AD	MRM						
Control	*DNMT3A*	2	25462012	G	A	NS	0.00001677	.	0.01	D	0	D	1	D	25.7	37	LB	MRM						
Control	*DNMT3A*	2	25467408	C	T	SSA	0.00003315	.	.	.	.	.	1	D	13.71	41	LB	MRM						
Control	*DNMT3A*	2	25462017	T	C	NS	0.00000837	.	0	D	0	D	1	D	23.8	44	LB	MRM						
Control	*DNMT3A*	2	25457242	C	T	NS	0.00050000	0.00070	0.03	D	0	D	1	D	23.1	51	LB	MRM						
Control	*DNMT3A*	2	25463583	G	A	NS	0.00001649	.	0	D	0	D	1	D	23.7	51	LB	MRM						
Control	*KDR*	4	55963895	C	T	NS	.	.	0.47	T	0	D	1	D	36	22	AD	MRM						
Control	*KMT2A*	11	118378293	T	G	NS	0.00000827	.	0.54	T	0.183	N	1	N	7.474	5	Cont	MRM						
Control	*TET2*	4	106158219	A	ATTTGA CCGCTC	FSI	.	.	.	.	.	.	.	.	.	18	AD	MRM						
Control	*TET2*	4	106164068	G	A	NS	.	.	0	D	.	.	1	D	22	20	AD	MRM						
Control	*TET2*	4	106190811	G	A	S	.	.	.	.	.	.	.	.	.	4	Cont	MRM						
Control	*TET2*	4	106182914	A	G	SSA	.	.	.	.	.	.	1	D	21.5	37	LB	MRM						
Control	*TET2*	4	106158372	A	AC	FSI	.	.	.	.	.	.	.	.	.	50	LB	MRM						
Control	*TET2*	4	106158509	G	A	NS	0.00003322	.	0.27	T	0.032	N	1	D	21.7	20	AD	MRM						
Control	*TP53*	17	7578526	C	T	NS	.	.	0	D	0	D	1	D	25.4	31	AD	MRM						
Control	*TP53*	17	7578503	C	T	NS	.	.	0	D	0.016	N	0.995	D	13.43	54	LB	MRM						

Supplementary Table 6. Mutational signatures. Comparison of the Pearson correlation product moment correlation coefficient values (r^2) between the 21 different mutational signatures observed in cancer as determined by Alexandrov et al [27], and the mutational signatures seen in both Single Regional Mutations (SRMs) and Multiple Regional Mutations (MRMs) in our study.

Mutational signature from Alexandrov et al	Focal mutation (r^2 value)	MRM (r^2 value)
Signature1A	0.617813	0.153022
Signature1B	0.5608662	0.2580154
Signature2	0.05990177	0.1291803
Signature3	-0.1655428	0.08372614
Signature4	-0.04539417	-0.07720455
Signature5	0.1114828	0.2058733
Signature6	0.6615663	0.1960743
Signature7	0.1259546	0.2942886
Signature8	0.1418824	-0.05672829
Signature9	-0.06170881	-0.1156328
Signature10	0.2350385	-0.04068971
Signature11	0.05486709	0.3558389
Signature12	-0.06881005	0.04614821
Signature13	0.01351007	0.03222178
Signature14	0.428101	0.2307443
Signature15	0.6327862	0.2275191
Signature16	-0.064039	0.02431248
Signature17	-0.01968848	-0.01370575
Signature18	0.006402309	-0.08938579
Signature19	0.1848091	0.2813491
Signature20	0.08050852	0.07071721
Signature21	0.01208709	0.08217952

Supplementary Figures

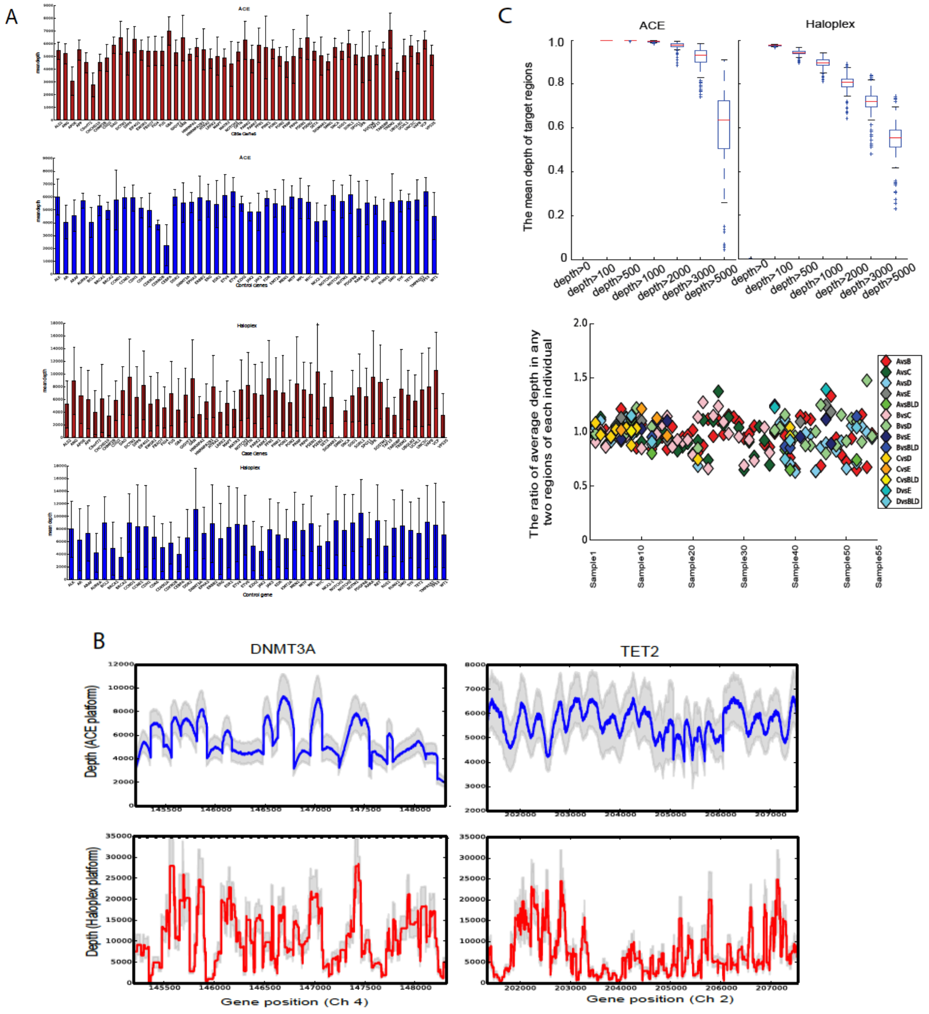

Supplementary Figure 1. Additional data on sequencing depth and coverage. (A) Mean coverage (+SD) for each neurodegenerative and cancer gene on both the ACE (Accuracy and Content Enhanced) and Haloplex^{HS} platforms across all samples. (B) Mean depth of the *DNMT3A* and *TET2* genes across all samples. Results using both the ACE capture kit (blue), together with the Haloplex^{HS} kit in red. The mean depth of sequencing at each base pair is shown together with the standard deviation (grey). Base positions can be seen along the X-axis with the means sequencing depth on the Y-axis. (C) Top – Box whisker plot of the proportion of variants covered per 1000-fold coverage change in depth on the ACE platform (left), and the Haloplex^{HS} (right). Bottom – The ratio of coverage depths between paired samples used for paired sample somatic calling. Each symbol represents a single air in a single individual. Key – A= Cerebellum, B=Entorhinal cortex, C= Frontal cortex, D=Medulla, E = Cingulate cortex.

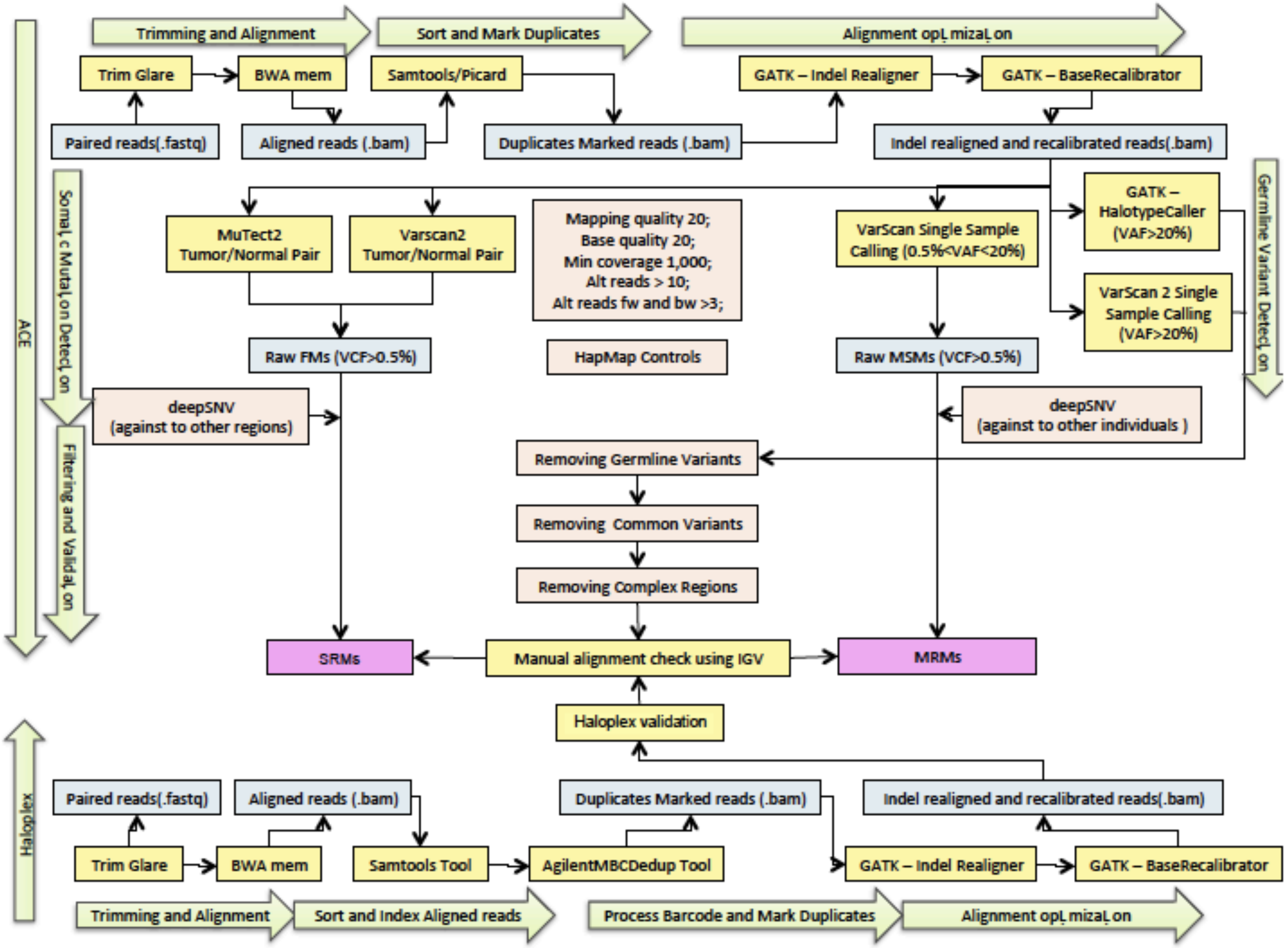

Trimming and Alignment
Sort and Mark Duplicates
Alignment opL mizaL on
Trim Glare
BWA mem
Samtools/Picard
GATK – Indel Realigner
GATK – BaseRecalibrator
Paired reads(.fastq)
Aligned reads (.bam)
Duplicates Marked reads (.bam)
Indel realigned and recalibrated reads(.bam)
ACE
SomaL c MutaL on DetecL on
MuTect2 Tumor/Normal Pair
Varscan2 Tumor/Normal Pair
Mapping quality 20; Base quality 20; Min coverage 1,000; Alt reads > 10; Alt reads fw and bw >3;
VarScan Single Sample Calling (0.5%<VAF<20%)
GATK – HalotypeCaller (VAF>20%)
VarScan 2 Single Sample Calling (VAF>20%)
Germline Variant DetecL on
Raw FMs (VCF>0.5%)
HapMap Controls
Raw MSMs (VCF>0.5%)
deepSNV (against to other regions)
deepSNV (against to other individuals)
Filtering and ValidaL on
Removing Germline Variants
Removing Common Variants
Removing Complex Regions
SRMs
Manual alignment check using IGV
MRMs
Haloplex validation
Haloplex
Paired reads(.fastq)
Aligned reads (.bam)
Duplicates Marked reads (.bam)
Indel realigned and recalibrated reads(.bam)
Trim Glare
BWA mem
Samtools Tool
AgilentMBCDedup Tool
GATK – Indel Realigner
GATK – BaseRecalibrator
Trimming and Alignment
Sort and Index Aligned reads
Process Barcode and Mark Duplicates
Alignment opL mizaL on

Supplementary Figure 2. Bioinformatic workflow. The work-flow for the ACE platform can be seen in the top portion of the figure moving down (identified on the left), and the Haloplex^{HS} pipeline from the bottom up. Green arrows indicate the process, yellow boxes - the bioinformatics programmes used, blue boxes - file outputs, pink boxes - methods. In summary, *ACE platform alignment and processing:* (1) Trimming of the raw paired-end reads (Trim Galore http://www.bioinformatics.babraham.ac.uk/projects/trim_galore/) with default settings. Three base pairs (bp) were removed from the 3' end of both paired reads after adapter/quality trimming has been performed. (2) Individual read groups were aligned to GRCh37 human genome by Burrows-Wheeler Aligner (BWA (v0.7.12)) [28]. (3) Aligned reads were indexed and sorted with Samtools (v1.3) [29]. (4) Duplicate reads were marked by Picard (v1.130) [30](https://github.com/broadinstitute/picard). (5) GATK (v3.5) [31, 32, 33] was used to recalibrate base quality scores and perform local realignment around known insertions and deletions. *Haloplex platform alignment and processing:* (1) As the HaloPlex^{HS} protocol uses a different vector adding an extra base to the adapter, this base was removed from the beginning of raw read 2. Both the read 1 adapter (GAGATCGGAAGAGCACACGTCTGAACTCCAGTCAC) and read 2 adapter (AGATCGGAAGAGCGTCGTGTAGGGAAAGAGTGT) were trimmed using Trim Galore. (2) Three base pairs (bp) were removed from both 3' end of paired reads and 5' end of paired reads after adapter/quality trimming, and individual read groups were aligned to GRCh37 human genome by Burrows-Wheeler Aligner (BWA (v0.7.12)). (3) Aligned reads were indexed and sorted with Samtools(v1.3). (4) AgilentMBCDedup (http://www.genomics.agilent.com/en/NGS-Data-Analysis-Software/AgilentMBCDedup-Tool/?cid=AG-PT-154&tabId=prod2510002) was used to process the Molecular Barcode (MBC) information of HaloPlex^{HS}, and functions to tag read pairs in a bam/sam file with their MBC sequences read out of the index 2 FASTQ files, and mark MBC duplicates from that sam/bam file. (5) GATK (v3.5) was used to recalibrate base quality scores and perform local realignment around known insertions and deletions. (6) BAM files were analysed for coverage depth QC using bedtools (v 2.25) [34] to calculate the depth per base and the histogram of coverage from the target regions using custom scripts. *Key:* BWA – Burrow Wheeler Aligner, GATK – Genome Analysis Tool Kit, SRMs –Single Regions Mutation, MRMs – Multiple Region Mutations, VCF – Variant Calling Format, VAF – Variant Allele Frequency.

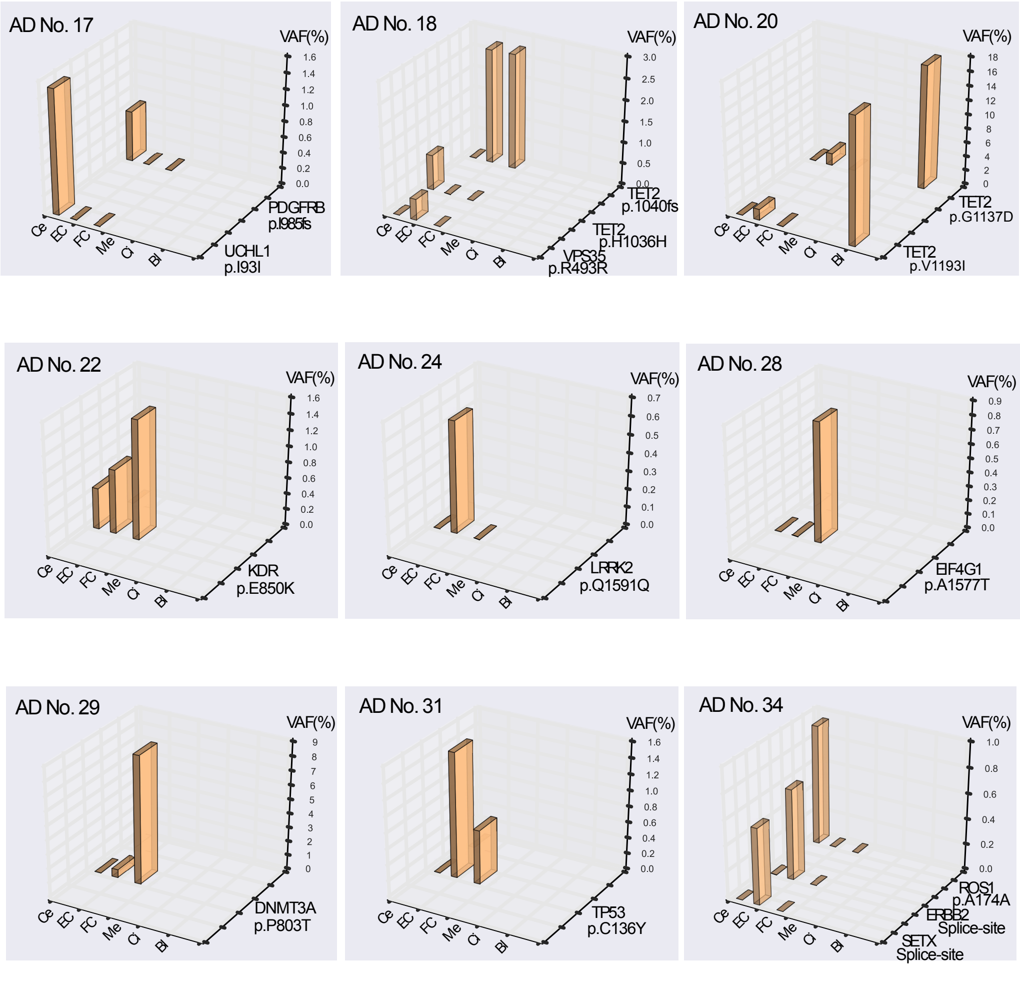

Supplementary Figure 3. Somatic mutations detected in cases with Alzheimer's disease (AD). Case numbers corresponding to those cited in the text and Supplementary tables 5 and 6. The brain region in which the mutation was observed in shown in the X axis, the gene and amino-acid change shown on the Y axis and the variant allele frequency shown on the Z axis.

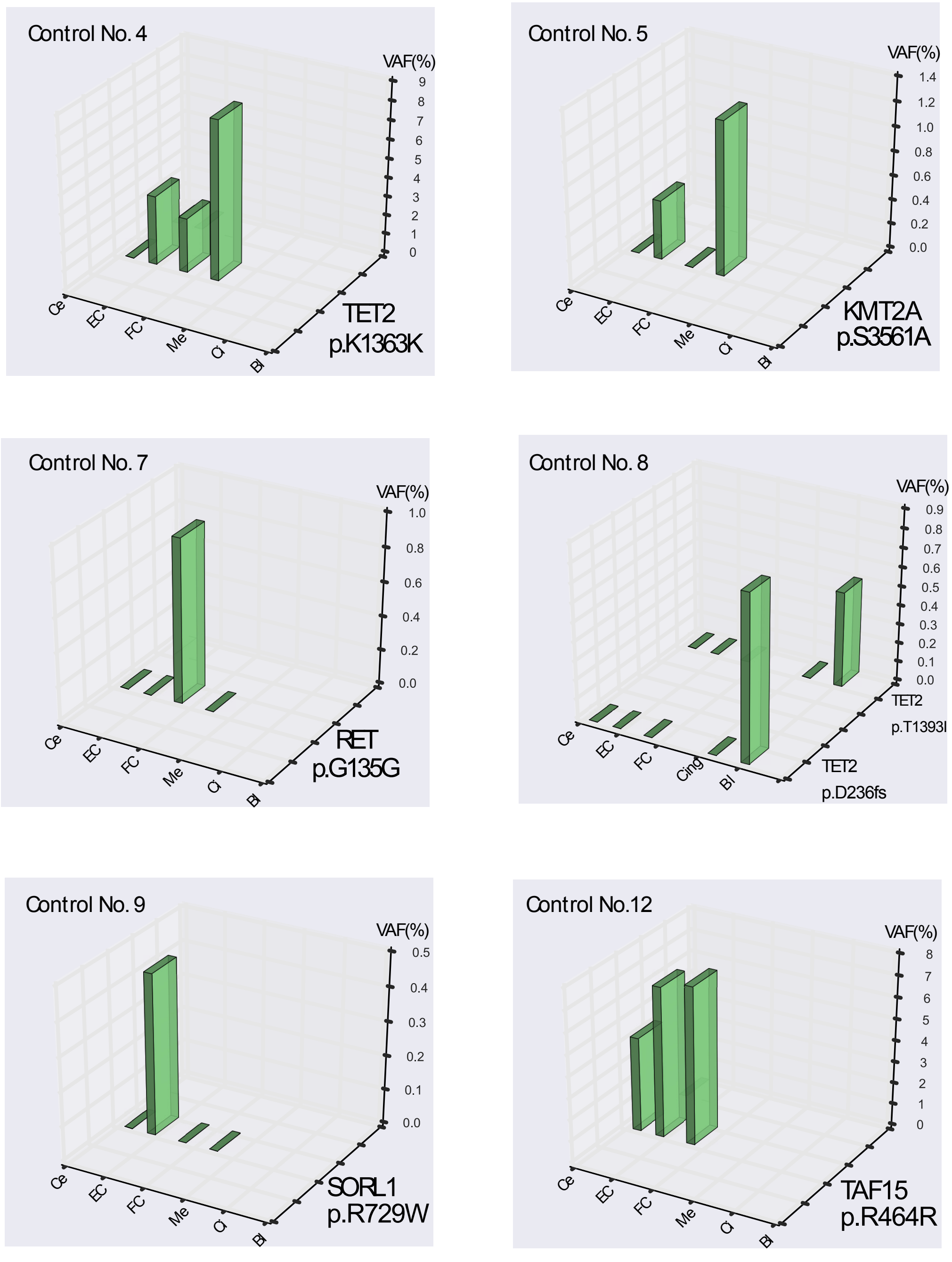

Supplementary Figure 4. Somatic mutations detected in control cases. Case numbers corresponding to those cited in the text and Supplementary tables 5 and 6. The brain region in which the mutation was observed in shown in the X axis, the gene and amino-acid change shown on the Y axis and the variant allele frequency shown on the Z axis.

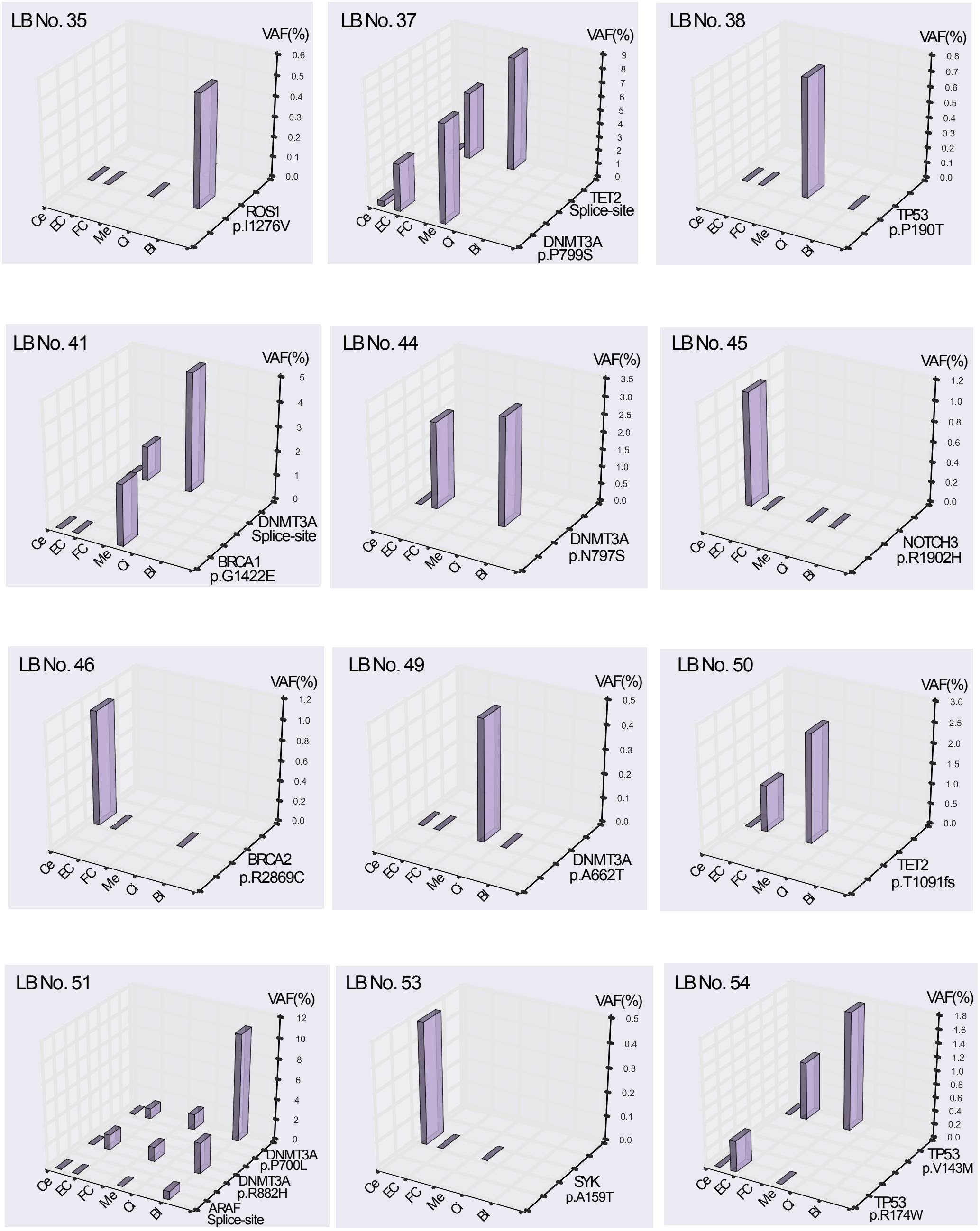

Supplementary Figure 5. Somatic mutations detected in individuals with Lewy body disease (LB). Case numbers corresponding to those cited in the text and Supplementary tables 5 and 6. The brain region in which the mutation was observed in shown in the X axis, the gene and amino-acid change shown on the Y axis and the variant allele frequency shown on the Z axis.

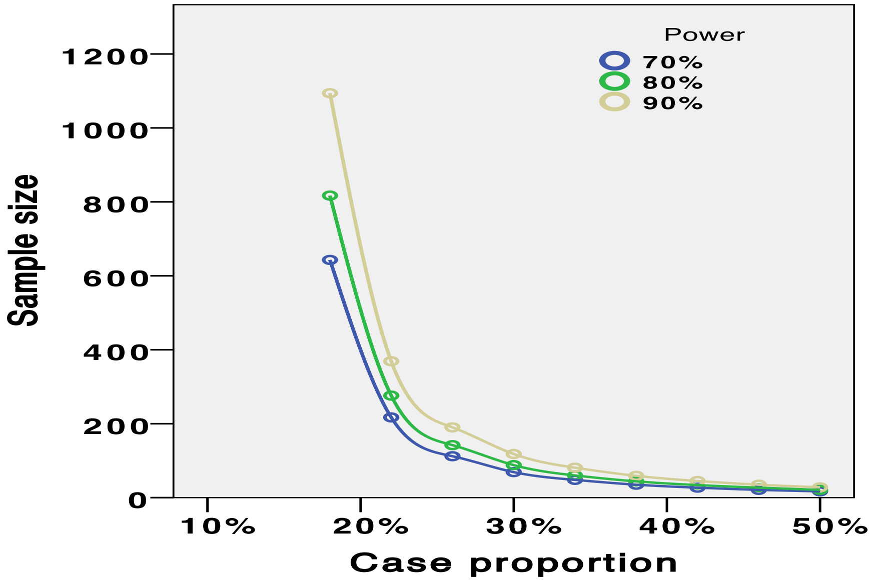

Supplementary Figure 6. Power curve for the number of samples required to see a difference between cases and controls. The number of case and control samples required to see a statistically significant ($P<0.05$) difference in overall focal somatic mutation rates in case neurodegenerative disease genes between a disease cohort and controls. Assuming a 13% carrier frequency in controls (based upon a 13% prevalence in all 57 cases studied to date), we calculate the required case and control numbers to see a range of increased carrier proportions in cases (as shown).

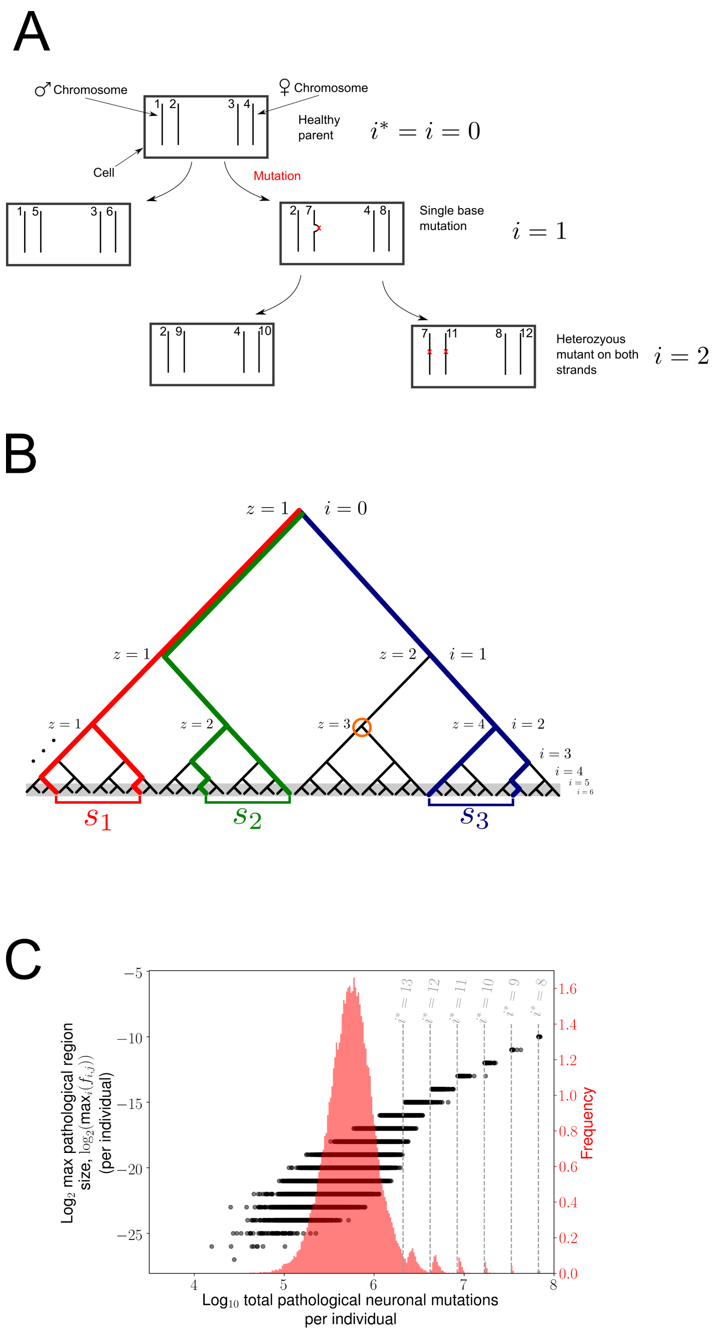

Supplementary Figure 7. Branching process model of neurodevelopment

(a) We idealise the cell as consisting of two chromosomes, one from each parent. We only consider the 132617 bp of the case genes. During division each strand is copied. To illustrate this, strands are labelled with integers and new strands are labelled with a new integer. Neurodevelopment is modelled to begin with a single founder cell, the generation number beginning at i = 0. Consider in this example a copying error which generates a SNV in strand 7 whilst copying strand 2 at

generation $i^* = i = 0$. Strand 7 is subsequently copied in the following generation to create a heterozygous mutant present on both strands. Thus, a mutation occurring during copying at generation $i^* = 0$ causes 1 in 4 daughter cells at generation i = 2 (and all subsequent generations) to harbour a heterozygous mutant. In general, the relative region size (f) may be expressed in terms of the mutant generation number as $f = 2^{-(i^*+2)}$. (b) Simulation strategy for approximate Bayesian computation of mutation rate. In this illustrative example, assume that the human brain consists of 2^6 cells and 3 samples (s_1, s_2 and s_3) of 11 cells are taken from the brain. We may assign integer labels to cells from left to right for a given generation number ($z \in [1,2^i]$ for integer z), giving each cell in the lineage a unique coordinate (z,i). Given the coordinates of the sampled cells, we only wish to simulate mutation events in the lineage of parents which give rise to sampled cells: these are the cells which lie on or between the coloured boundaries. For instance, at generation i = 1, we must simulate mutations for both cells z = 1 and z = 2. However, cell (3,2) (circled) has no influence over the sampled cells, and therefore does not need to be simulated. Cells in the final generation (i = 6 in this case) form the adult brain and are not replicated. Replication of cells in the penultimate generation cannot give rise to mutants present on both strands of DNA (see a), and therefore may also be neglected (grey shaded region) since mutations were checked for their presence on the reverse strand. Thus, in this example, we need only consider cells with $i \leq 4$. (c) Multimodality in the distribution of total pathological mutations per individual is driven by the largest pathological region per individual. Correlation between the largest pathological region over all mutations i, for individuals j, and the total number of pathological mutations per individual (grey point for each individual), overlaid with the distribution of total pathological mutations per individual. The number of cells corresponding to regions seeded at generation i^* = 8,9,…,13 are shown in grey. At fixed i^*, e.g. i^* = 11, for individuals whose largest mutant region occurred at i^* = 12 (corresponding to $log_2(f)$ = −13, see (a)), the width of the corresponding mode of the distribution of total pathological mutations is small relative to the number of mutant cells associated with e.g. mutations seeded at i^* = 12. Hence, individuals with a mutation seeded at i^* = 12 have sufficiently more pathologically mutated cells to induce a separate mode.

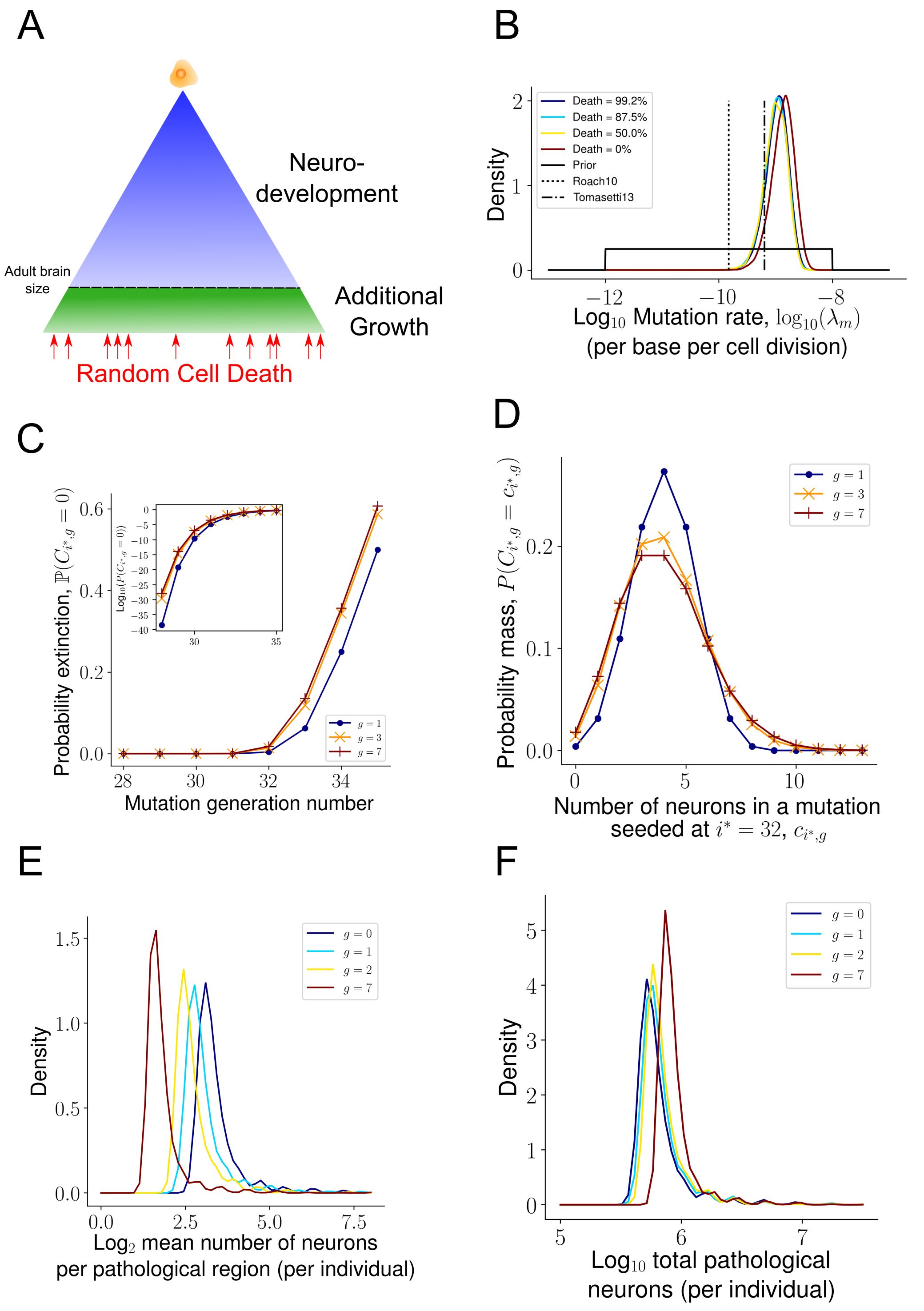

Supplementary Figure 8. Investigation of a simple model of cell death during neurodevelopment. (a) We model cell death during neurodevelopment by allowing additional generations of growth (g = 1,2, ...) (green shaded), with stochastic cell death at the end of the additional growth (red arrows) such that the final number of cells is the number of cells in the adult human brain. (b) The inferred mutation rate was found to be broadly unchanged for g = 1,2,7 relative

to the original model without cell death ($g = 0$). (c) The probability of extinction of a mutant region via cell death becomes negligible for mutation generation numbers $i^* \leq 32$. Inset displaying the same plot on a logarithmic scale for the vertical axis. (d) Distribution of the number of cells in a mutant region seeded at $i^* = 32$ for differing levels of cell death. (e) Mean number of neurons per pathological region for differing levels of cell death. (f) Total number of pathological neurons per individual for differing levels of cell death. In (e-f), we assume that mutant regions are diffuse in the adult brain.

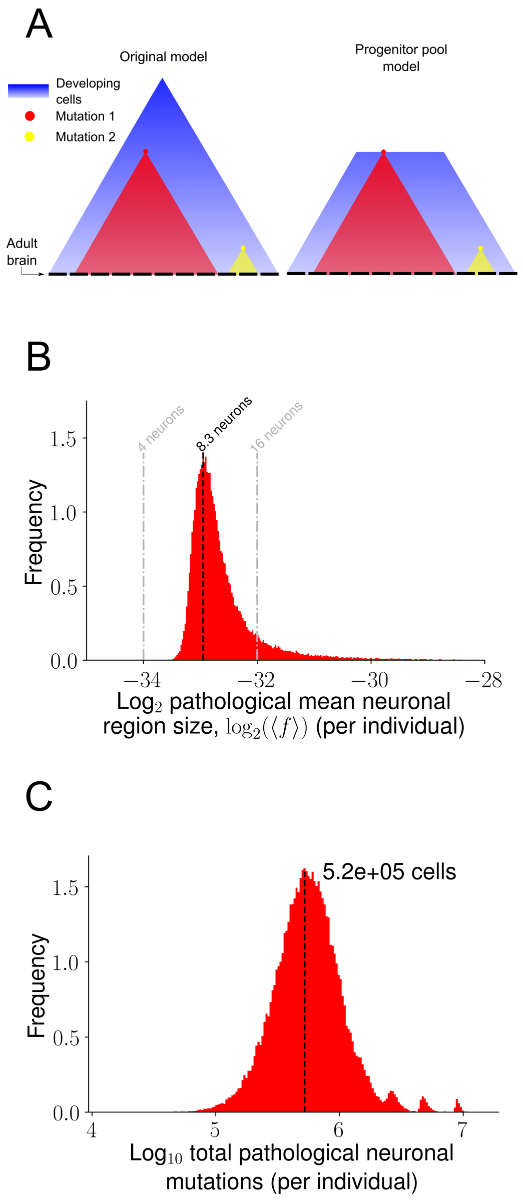

Supplementary Figure 9. Investigation of the progenitor pool model. (a) In the progenitor pool model, neurodevelopment is assumed to begin with 210 = 1024 unmutated cells, which is followed by subsequent rounds of symmetric cell division. Note that, by construction of the model, a mutation which affects e.g. one of the progenitors in the progenitor pool model (red dot, right) has the same mutant region size in the final adult brain as a mutation occurring at generation 10 in the original model (red dot, left). This is the case for all mutations after generation 10 (e.g. yellow dots) (b) The mean pathological region size is almost unchanged relative to the original model (see Fig. 5d). (c) The total number of pathologically mutated neurons per individual is almost unchanged relative to the original model.

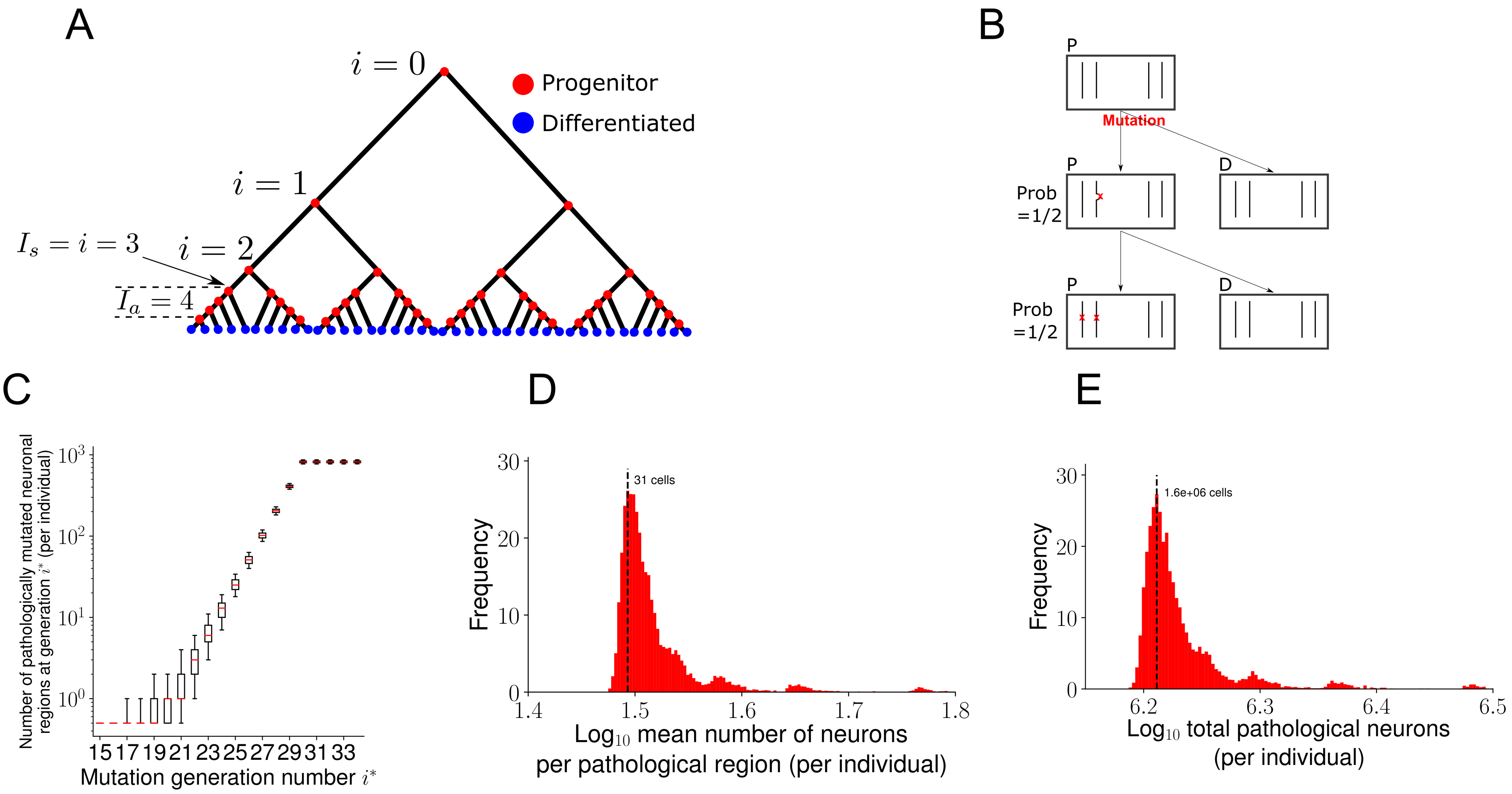

A
$i = 0$
Progenitor
Differentiated
$i = 1$
$I_s = i = 3$
$i = 2$
$I_a = 4$
B
P
Mutation
P
D
Prob =1/2
P
D
Prob =1/2
C
Number of pathologically mutated neuronal regions at generation i^* (per individual)
10^0
10^1
10^2
10^3
15 17 19 21 23 25 27 29 31 33
Mutation generation number i^*
D
31 cells
Frequency
0
10
20
30
1.4
1.5
1.6
1.7
1.8
Log_{10} mean number of neurons per pathological region (per individual)
E
1.6e+06 cells
Frequency
0
10
20
30
6.2
6.3
6.4
6.5
Log_{10} total pathological neurons (per individual)

Supplementary Figure 10. Investigation of a simple asymmetric model of neurodevelopment. (a) Neurodevelopment is modelled as consisting of a phase of symmetric division, followed by a phase of asymmetric division. (b) Asymmetric division induces a stochastic element to the inheritance of a mutation. Showing an example of a mutation event where a copying error occurs in a progenitor (P) cell (top) which is inherited by a daughter progenitor cell (middle); this occurs with probability 1/2. The single-stranded mutation is copied, and a double-stranded mutation is inherited by another progenitor cell (bottom) (probability 1/2). This mutation, which is now on both strands of DNA, will be inherited by all subsequent daughters (both P and D). Either of the preceding events could have been inherited by differentiated cells (D), which do not divide, and therefore affect a greatly restricted number of cells. (c) The distribution of pathologically mutated neuronal regions initially increases exponentially until the asymmetric division phase at generation 30, where the number of mutant regions remains constant thereafter. (d) Mean number of mutated neurons per mutated region, with a mode of 31 cells. (e) Total number of mutations per individual, with mode of 1.6×10^6 cells.

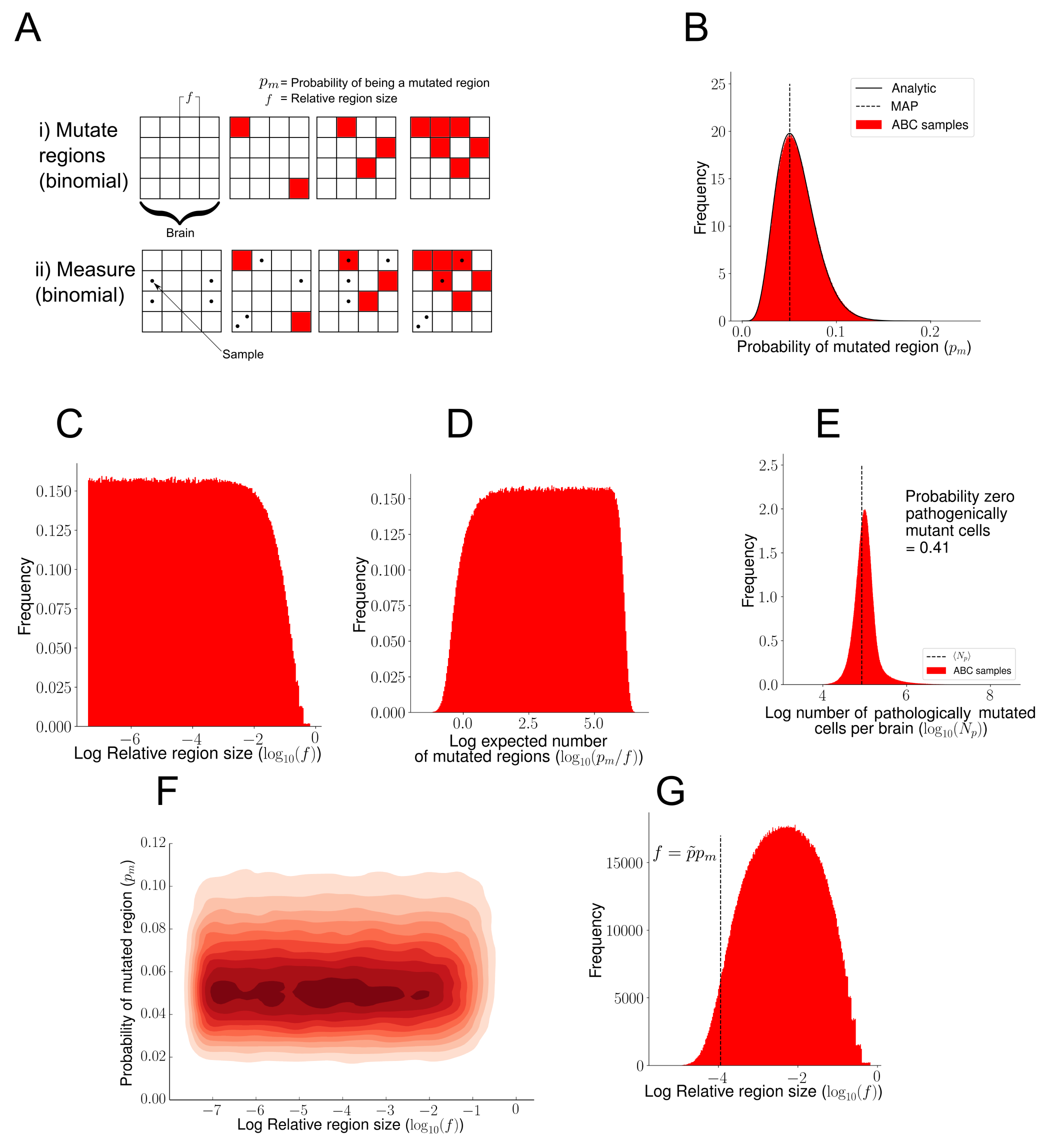

Supplementary Figure 11. Constant region size model of neurodevelopment. (a) We use a simplified model of mutagenesis in the brain, where the brain consists of independent 'regions' of relative size *f*, where *f* is an unknown constant. Each region (small square) has an independent probability of being homogeneously mutated in any of the case genes (p_m, red square), with the number of mutated regions being binomially distributed. We model the measurement process as each sample (black circle) being an independent trial, with the number of samples being positive for a mutation as binomially distributed. (b) The marginal posterior distribution of p_m is constrained by the data to be p_m = 0.024–0.11 (95% B.C.I.). Displaying the analytic posterior

distribution if p_m is treated independently of f (solid black line), and the maximum a posteriori estimate (MAP) $(\mathrm{p_m})_{\mathrm{MAP}}$ = 6⁄119 (dotted black line). This is supported by the lack of covariation observed between p_m and f (see (f)). (c) The marginal posterior distribution of f is not strongly constrained by the data, with f = 5.7 × 10^{-8}–0.11 (95% B.C.I.), although support reduces markedly as $f \rightarrow 1$. (d) However, the mean number of mutated regions per individual ($\langle x \rangle = p_m/f$) is constrained strongly enough such that the lower bound of $\langle x \rangle \approx 1$ ($\langle x \rangle$ = 0.942 ± 6.6 × 10^{-3}, 5th percentile of approximate posterior ± error. The assigned error was determined by splitting the posterior samples into 10 subsets of equal size, and taking the sample standard deviation of the 5th percentile of each subset). This means that individuals have, on average, at least ∼ 1 mutant region, but potentially many more. (e) Using the probability that a region is pathologically mutated ($\tilde{p}$= 298 ⁄132617 = the number of pathological SNVs across the case genes), the number of pathologically mutated neurons per individual may be simulated. 37% of simulated individuals had zero pathologically mutated cells (corresponding to when $f \gg \tilde{p}p_m$ (see a)), but those that did tended to have ∼ 10^5 mutated neurons. $\langle N_p \rangle$ = 1.7 × 10^5 (black dotted, see Mathematical Supplement for derivation), which is in good agreement with Fig. 5E. (f) Joint distribution of p_m and $log_{10}(f)$ shows that p_m and f are well-approximated as being independent. (g) Panel (e) shows that 41% of simulated individuals had zero pathologically mutated cells. For these individuals, we may plot the posterior distribution of relative region sizes, i.e. $P(\log_{10}(f)|N_p = 0, \varrho(S_m(\mathrm{D}), S_d(\mathrm{D})) = 0))$. The support decays when $f \ll \tilde{p}p_m \approx \tilde{p}p_m$=1.7 × 10^4 (black dotted line). This is the limit that the mean number of pathologically mutated regions per individual is much less than 1, i.e. $\langle \tilde{Y} \rangle \ll 1$, where $\tilde{Y}$ is defined in Eq.(40) of the Mathematical Supplement.

Supplementary Methods

Approximate Bayesian computation for crude branching model of neurodevelopment

The genetic information we have allows us to make preliminary statements about the number of cells that might be affected by pathological mutations. The objective here is to place the empirical observations in a simplistic theoretical context, not to provide a definitive set of estimates. For more definitive estimates, more refined models, inference and data would be required. That noted, the inference in Fig. 5B, and outlined below, can be decoupled from Figs. 5C-H by simply using literature estimates for the mutation rates. The conclusions we make do not then change qualitatively if we use appropriate literature values in Figs. 5C-H. Of course, Figs. 5C-H do still depend on the appropriateness of our very simple model, see Supplementary Note 1–Supplementary Note 5.

We consider neurodevelopment as a deterministic branching process where every cell has exactly two daughters per generation and the probability of death is zero (see Supplementary Note 1 for an investigation of non-zero cell death). We assume that the brain begins with a single founder cell (see Supplementary Note 2) which doubles exactly 37 times, yielding $2^{37} \approx 1.37 \times 10^{11}$ cells in the adult brain (both neuronal and non-neuronal cells). We use an integer number of doublings for computational convenience, and chose 2^{37} as the closest number of cells to experimental measurements of the adult brain, being approximately $1.7 \pm 0.14 \times 10^{11}$ cells ($\pm$ SD).[35] This is a slight underestimate of the true number of neurons in the adult brain (falling 2.4 SD below the mean, 19% difference in mean estimates). After inferring the mutation rate given the data (see below), we will seek to generate estimates of the number of pathologically mutated neurons in the adult human brain. We do this by considering the development of just the neuronal population in the same manner as above, except with 36 doublings yielding $2^{36} \approx 6.9 \times 10^{10}$ neurons. This falls within 2 standard deviations of the estimate of the number of neurons in the brain by Azevedo *et al.* ($86.1 \pm 8.1 \times 10^9$ neurons), and is therefore compatible with that study.

Our simulation proceeds by sampling a mutation rate (λ_m, per base per cell division) from a prior distribution, which was motivated by the literature. A study by Roach *et al.* involved family-based whole genome sequencing in peripheral blood cells to deduce the human intergeneration mutation rate as $\sim 1.1 \times 10^{-8}$ per bp per generation per haploid genome.[36] In order to approximate the mutation rate per base per cell division, we estimate the number of cell divisions per generation to form the adult brain as 36 (given our model). Since both strands of DNA are replicated during cell division, we estimate $\lambda_m \approx 1.1 \times 10^{-8}/(2 \times 37) \approx 10^{-10}$ per base per cell division. However, this order of magnitude estimate is subject to large uncertainty, since it is unclear the extent to which the germline mutation rate is representative of the mutation rate during neurodevelopment. Consequently, we chose to search ± 2 orders of magnitude around this estimate, using the following broad uninformative prior on λ_m:

$$P(\ln(\lambda_m)) = \text{unif}(\ln(10^{-12}), \ln(10^{-8})) \quad (1)$$

where unif(a, b) is a uniform distribution between a and b. Note that the prior of λ_m is uniform on log-space, and therefore encodes our belief that λ_m is uncertain over 4 orders of magnitude

Given a sample of the mutation rate from the prior, we are then able to simulate neurodevelopment for an individual. Our strategy will be to simulate 40 such individuals (the number of individuals in the case cohort) and, loosely speaking, if a simulation is 'close enough' to the data we will accept the draw from the prior as a sample from the approximate posterior distribution for λ_m, which captures all of our uncertainty in the parameter given the data. This is the essence of approximate Bayesian computation (ABC).

We note that we restricted our attention to the case cohort (consisting of 40 individuals) and neglected the control cohort (14 individuals). Although there is insufficient evidence to reject the

null hypothesis that both patient and controls have different propensities towards single-regional mutation (see Main Text), this is not the same as positive evidence for both groups having the same mutation rate. For simplicity, we restrict our attention to the larger case cohort, but the similar frequencies of mutation across these groups provides an initial suggestion that our findings could generalise to wider populations.

The way in which tissue samples map to the simplistic tree structure of our model of the brain is clearly non-trivial. We assume that all samples consist of 3415 cells, which is the experimentally-determined average number of captured cells per sample. We make the strong assumption that the sampled cells are spatially contiguous, and developmentally similar, and may therefore be modelled as a contiguous 1D embedding in the leaves of the tree (see Fig. 5A and Supplementary Figure 7B). The assumption of spatial contiguity of the sampled cells enhances computational tractability of the inference approach described below and is discussed in more detail in Supplementary Note 4.

In Supplementary Figure 7B we show that it is only necessary to consider the set of parent cells which give rise to the cells in the sample (given the assumption of spatial continuity of the sampled cells, see above). Once the set of parental cells have been determined, we seek to compute the locations of mutation events in the lineage. Given a draw of the mutation rate λ_m from the prior (Eq.(1)), we assume that each base has a probability λ_m of mutating during replication. The total number of bases which are copied during development (B) is $B = 4N_bN_p$ where N_b is the number of base pairs to be considered (132617 bp in the case genes) and N_p is the number of parent cells which give rise to the sampled cells. The factor of 4 counts for the ploidy of the human genome (2 genomes) and the number of strands in DNA (2 strands) (see Supplementary Figure 7A). The total number of mutation events which occur in the lineage (M) is therefore modelled to be

$$P(M) = \mathrm{Binom}(B, \lambda_m) \tag{2}$$

where $\mathrm{Binom}(N, p)$ is the binomial distribution with N trials and probability of success p.

In order to find the locations of mutation events in the lineage, $R_1, R_2, \ldots, R_m$, we wish to sample from the joint distribution $P(R_1 = r_1, R_2 = r_2, \ldots, R_m = r_m, M = m)$ where upper case variables are random variables and lower case variables are realizations of the corresponding random variable. Using the product rule of probability, $P(R_1 = r_1, \ldots, R_m = r_m, M = m) = P(R_1 = r_1, \ldots, R_m = r_m|M = m)P(M = m)$. $P(M = m)$ is given in Eq.(2). Since we have assumed that each base is equally likely to mutate during division, the conditional distribution is assumed to be

$$P(R_1 = r_1, \ldots, R_m = r_m|M = m) = \Pi_{k=1}^{m}\mathrm{unif}\{1, B\} \tag{3}$$

where $\mathrm{unif}\{a, b\}$ denotes the discrete uniform distribution over $[a, b]$ (note an analogous result is provably true in continuous space for Poisson-distributed events[37]). Thus, to sample from the joint distribution, we first draw a Binomial random variable for the total number of mutation events, and the location of each mutation event will be uniformly distributed across all of the bases in the lineage.

In doing this, we are assuming that mutations never affect the same cell at the same base more than once (although the same mutation may affect multiple different cells). This assumption is justified with the following calculation. Throughout the lineage history each base is assumed to be copied 36 times. If we assume a worst-case mutation rate of the upper-bound of our prior in Eq.(1), $\lambda_m = 1 \times 10^{-8}$, then the total number of times a single base is mutated is $X \sim \mathrm{Binom}(37 - 2, \lambda_m)$ (the factor of (-2) is due to considerations discussed in Supplementary Figure 7A). It follows that $P(X \geq 2) = 5.9 \times 10^{-14}$. We thus calculate an upper bound on the total number of times bases are mutated more than once across the entire brain of an individual by assuming that every base in every cell has an independent chance of being mutated, and therefore the total number of such events is $2^{37} \cdot 4N_b \cdot P(X \geq 2) \approx 4337$ multiple-mutated bases per individual. This is a strong overestimate since neurons share lineage history, so in effect the factor of 2^{37} is actually much lower. Given that

the total number of mutation events in the brain is on average $4N_b \cdot \lambda_m \cdot \sum_{k=0}^{35} 2^k \approx 3.6 \times 10^8$ mutations per individual then, even for this overestimate, bases being mutated more than once per cell is an extremely rare event ($\mathcal{O}(10^{-5})$ of mutation events) and is thus neglected.

A mutation which occurs at generation i^* affects a fraction (f) of the brain, where

$$f = 2^{-(i^*+2)}. \tag{4}$$

The factor of (+2) arises from the lag associated with copying a single-stranded mutation into a daughter cell to become a double-stranded mutation, see Supplementary Figure 7A. Using this observation, once the position of mutations in the lineage have been determined using Eq.(2) and Eq.(3), the overlap between the daughters of the parental mutant cell and the sample can be determined to find the total number of mutations in sample s from patient p from mutational event j (given the assumption of spatial continuity of the sampled cells, see above), in the cohort denoted as $X_{j,p,s}$. Note that $\sum_j X_{j,p,s}$ is not necessarily equivalent to the total number of mutated cells per sample, since a single cell may harbour multiple different mutations (e.g. consider the case of the founder cell of the brain containing a mutation, as well as one of its daughters containing a different mutation, then a subset of the final population of cells harbour two mutations per cell).

To determine whether the sample from the prior λ_m may be accepted as a sample from the approximate posterior distribution, we use summary statistics to compare the simulation with data ($\mathcal{D}$). Experimentally, the threshold for detection of a SNV per sample was $\sim 0.5\%$ VAF. Using an average sample size of 3415 cells, this corresponds to a detection threshold of ≥ 17 cells in order for a mutation to be detectable amongst the captured cells of the sample. Before defining the summary statistics, we define the following indicator functions

$$\Phi(x) = \begin{cases} x \text{ if } x \geq 17 \\ 0 \text{ otherwise} \end{cases} \tag{5}$$

$$I(x) = \begin{cases} 1 \text{ if } x > 0 \\ 0 \text{ otherwise} \end{cases} \tag{6}$$

where $\Phi(x)$ is based on the experimental detection threshold. The three summary statistics $\mathbf{S} = (\Gamma_1, \Gamma_2, \Gamma_3)$ of the model are defined as:

$$\Gamma_1 = \sum_{p=1}^{40} \sum_{s=1}^{s_p} \sum_{j=1}^{M} \Phi(X_{j,p,s}) \tag{7}$$

$$\Gamma_2 = \sum_{p=1}^{40} I\left(\sum_{s=1}^{s_p} \sum_{j=1}^{M} \Phi(X_{j,p,s}) \right) \tag{8}$$

$$\Gamma_3 = \frac{1}{\sum_p \sum_s \sum_j I(\Phi(X_{j,p,s}))} \sum_p \sum_s \sum_j \frac{\Phi(X_{j,p,s})}{3415} \tag{9}$$

where s_p is the number of samples taken for patient p. Γ_1 measures the total number of detectable mutations across all samples and all patients, Γ_2 measures the total number of patients with any detectable mutations and Γ_3 is the average VAF for all detectable mutations across all samples and patients. Using a hat to denote data, these summary statistics were found experimentally to be:

$$\hat{\Gamma}_1 = 6 \tag{10}$$

$$\hat{\Gamma}_2 = 6 \tag{11}$$

$$\hat{\Gamma}_3 = 0.87 \times 10^{-2}. \tag{12}$$

Thus, the acceptance criteria we use are

$$|\Gamma_1 - \hat{\Gamma}_1| \leq \epsilon_{sa} \tag{13}$$
$$|\Gamma_2 - \hat{\Gamma}_2| \leq \epsilon_{pa} \tag{14}$$
$$|\Gamma_3 - \hat{\Gamma}_3| \leq \epsilon_{v} \tag{15}$$

where $\epsilon_{sa} = \epsilon_{pa} = 1$ and $\epsilon_v = 2\text{SEM}_v$ (SEM_v = standard error in the mean for the VAF across all positive experimental samples), and we demand that all three of the above criteria must be satisfied simultaneously. Given that these criteria are met, we accept λ_m as a draw from the approximate posterior distribution $P(\lambda_m|\rho(\mathbf{S}, \mathcal{D}))$ where ρ denotes the acceptance criteria given above. It is possible that a more compact and/or optimal set of summary statistics could be obtained.

Using the draws from the approximate posterior distribution, we may then forward-simulate each individual using a single-draw from $P(\lambda_m|\rho(\mathbf{S}, \mathcal{D}))$. We note that the distribution of inferred mutation rates is compatible with the mutation rate found by Tomasetti *et al.*[38] (see Fig. 5B), and therefore the extrapolations presented in Figs. 5C-H are likely to be robust given this literature value for the mutation rate. As we are interested in the prevalence of pathologically mutated neurons, rather than general brain cells, we use 36 instead of 37 generations as discussed above. We may therefore determine the distribution of mutant neuronal region sizes and the number of mutated neurons per individual (see Fig. 5). The number of mutations that arise at generation i^* (X_{i^*}) is

$$X_{i^*} \sim \text{Binom}(4N_b \cdot 2^{i^*}, \lambda_m) \tag{16}$$

where the number of trials is the total number of bases across all cells at generation i^* (see Fig. 5C).

With this result, we may determine the probability of observing one or more regions seeded at generation i^*, given our posterior estimate for λ_m (see Fig. 5F). We define Q_{i^*} as the proportion of N individuals with one or more mutated regions from generation i^*. Relabelling $\lambda_m \equiv \lambda$, and indexing each individual by j, corresponding to a single draw from the approximate posterior $\lambda_j \sim P(\lambda_j|\rho(\mathbf{S}, \mathcal{D}))$, we are able to simulate as many individuals (N) as we have available samples from the approximate posterior ($N \approx 10^5$). We may therefore write

$$Q_{i^*} = \frac{1}{N}\sum_{j=1}^{N} I(X_{i^*,j}), \tag{17}$$

which is the approximate posterior mean of the indicator function. We may then determine the standard deviation of Q_{i^*}. Observing that

$$\mathbb{E}(I(X_{i^*,j})) = 0 \cdot P(X_{i^*,j} = 0) + 1 \cdot P(X_{i^*,j} = 1) + 1 \cdot P(X_{i^*,j} = 2) \ldots \tag{18}$$
$$= 1 - P(X_{i^*,j} = 0) \tag{19}$$

(where $\mathbb{E}()$ denotes an expectation) it follows that, since all $X_{i^*,j}$ are independent,

$$\mathbb{E}(Q_{i^*}) = \frac{1}{N}\sum_{j=0}^{N}(1 - P(X_{i^*,j} = 0)) \tag{20}$$

$$\mathbb{V}(Q_{i^*})^{1/2} = \left[\frac{1}{N^2}\sum_{j=0}^{N}\left\{(1 - P(X_{i^*,j} = 0) - [1 - P(X_{i^*,j} = 0)]^2\right\}\right]^{1/2} \tag{21}$$

where $\mathbb{V}()$ denotes the variance and

$$P(X_{i^*,j} = 0) = (1 - \lambda_j)^{4N_b 2^{i^*}}. \tag{22}$$

Note that the standard deviation of Q_{i^*} scales as $1/N$ due to uncertainty arising from sampling noise in λ_j which diminishes as more samples from the approximate posterior distribution are obtained. Also note that the number of bases used in Eq.(22) is $N_b = 298$, as this is the number of pathological bases in the case genes. An empirical estimate of the standard error may be obtained by treating each individual as a Bernoulli trial with probability $p = P(X_{i^*} \geq 1)$, and SEM $\sqrt{p(1-p)/N}$. These are compatible with our uncertainty in the approximate posterior mean of the indicator function, shown in Eq.(21).

The visual representation in Fig. 5H was created by taking the mean of the approximate posterior samples for the mutation rate $\mathbb{E}(\lambda_m|\mathcal{D})$ as the mutation rate of the individual. The number of mutated neuronal regions for $20 \leq i^* \leq 27$ were drawn using Eq.(16), where $X_{i^*=20}$ was redrawn until $X_{i^*=20} \geq 1$. We think this is fair for illustrative purposes since Fig. 5F shows that $\sim 79\%$ of all simulated individuals possess at least one region seeded at generation $i^* = 20$. Each circular mutated region has an area proportional to the number of pathologically mutated neurons in the corresponding region: $k\pi r^2 = 2^{36} \cdot 2^{-(i^*+2)}$ where k is chosen arbitrarily. The radius and angle of each region relative to the centre of the whole brain was chosen via a uniform random distribution. Although the relative sizes of each region are to scale, the area of the whole brain is certainly not to scale with the mutated regions.

5×10^7 ABC iterations were performed, all code was written in `Python`, and is available from the authors upon request.

Supplementary Notes

Supplementary Note 1

Branching model and cell death

In the crude branching model of neurodevelopment, we neglected cell death for simplicity. However, programmed cell death plays an important role in neurodevelopment (reviewed in Ref. 39). We made the following modification to the model presented in the Main Text as a preliminary means of investigating the extent to which programmed cell death may influence our estimations. We note that the following model of cell death is, itself, a marked simplification of real cell death during neurodevelopment, but benefits from its parsimony and computational tractability. We again emphasize that for more definitive estimates, more refined models, inference and data would be required.

It is thought that 20-80% of neurons are lost during neurodevelopment, and that this wave of cell death often occurs at a relatively late stage in maturation.[40] To incorporate cell death into our model, and account for uncertainty in the level of cell death during development, we allow the brain to double in size for an additional number of generations, $g = 1, 2, \ldots,$ resulting in a brain which is a factor of 2^g larger than a normal adult brain; we will term this a "super-brain". We then infer the mutation rate by taking a sample from the super-brain which is a factor of 2^g larger than the experimental sample size (3415 cells); we term this a "super-sample". Note that we retain the assumption of spatial contiguity of the cells of the super-sample from the original model, for computational tractability. We then sample a fraction of $1/2^g$ cells from the super-sample (without replacement) as cells which survived to adulthood. In doing this, we always sample 3415 cells, but the spatial location of these cells within the super-sample is random. In sampling the tree after a phase of additional growth, we are effectively placing cell death at the leaves of the tree: an approximation which is more appropriate assuming that the bulk of cell death occurs late in development. We then proceed with the approximate Bayesian computation as discussed in Supplementary Methods. Consequently, this results in a model where a fraction $(1 - 1/2^g)$ of all cells die (see Supplementary Figure 8A). By computing the posterior distribution of the mutation

rate λ_m for different values of g, we may investigate the robustness of the mutation rate to this simple model of cell death.

We investigated values of $g = 0, 1, 2$ and 7 for death rates of 0% (Main Text model), 50%, 87.5% and 99.2% respectively. In Supplementary Figure 8B, we show that the approximate posterior distributions for the mutation rate have large overlap for different magnitudes of cell death. This result has an intuitive explanation given the following two observations about the model. Firstly, a mutation occurring at generation i^* affects a *fixed proportion* of the brain ($f = 2^{-(i^*+2)}$), regardless of how large the brain subsequently becomes. Secondly, we grow the brain to be a factor of 2^g larger than a normal adult brain, but use a survival rate of $1/2^g$ to ensure that number of cells which survive to adulthood is always the number of cells observed in a real adult brain, on average. Since the fraction of the brain that is mutated, and the total number of cells in an adult brain, are invariant to g, we would expect that the total number of cells which are mutated in an adult brain to be invariant to g. It is therefore unsurprising that the inferred values of λ_m are broadly invariant to g (and therefore are all compatible with the mutation rate from Tomasetti *et al.*[38]). The distributions are not identical because our crude model of cell death affects the size of mutational foci. Although the mean size of mutational foci given non-zero cell death is equivalent to the original, deterministic, case (see Eq.(25) below) these foci may fluctuate in size, as will be investigated below.

Given that the mutation rate is broadly invariant to the cell death rate, we suggest that many of our extrapolations to the human population (Figs. 5C-G) will also remain broadly invariant to this model of cell death. Whilst it is not straightforward to show this numerically, we provide some arguments for why this might be the case.

Firstly, consider a mutation seeded at generation i^*, which gives rise to $N_{i^*,g}$ daughter neurons prior to the cell death model, where

$$N_{i^*,g} = 2^{-(i^*+2)} \cdot 2^g \cdot N_{\text{neu}} \tag{23}$$

where N_{neu} = number of neurons in the brain $\approx 2^{36}$. When cell death is initiated in the model, N_{neu} cells are sampled without replacement from the super-brain of size $2^g N_{\text{neu}}$. These are the cells which survive to adulthood. We let $C_{i^*,g}$ be the number of surviving mutant daughter neurons seeded at generation i^*. $C_{i^*,g}$ may be modelled with a hypergeometric distribution. If $Z \sim \text{Hypergeom}(n, N, K)$, then Z is a random variable whose value is the number of successes in n draws taken without replacement from a population of size N where K objects have the feature of interest. Hence,

$$C_{i^*,g} \sim \text{Hypergeom}\left(N_{\text{neu}}, 2^g N_{\text{neu}}, 2^g N_{\text{neu}} 2^{-(i^*+2)}\right). \tag{24}$$

Using the properties of the hypergeometric distribution, the mean number of neurons which survive to adulthood is

$$\mathbb{E}(C_{i^*,g}) = N_{\text{neu}} 2^{-(i^*+2)}, \tag{25}$$

which is independent of g, as expected. This is equivalent to the number of mutations in the adult brain associated with a mutation event at generation i^* in the Main Text model.

Given the above argument, we now examine each extrapolation in Figs. 5C-G and explore their robustness to this simple model of cell death:

Number of pathologically mutated neuronal regions seeded at generation i^* (Fig. 5C)

We expect, according to our crude model of death and replication, that the distribution of the number of pathologically mutated neuronal regions seeded at generations $i^* \leq 32$ (X_{i^*}, see Eq.(16) and Fig. 5C) remains unchanged. The dynamics of neurodevelopment under our crude cell death model are equivalent up to generation 36. These dynamics are determined by a single parameter, the mutation rate λ_m, the inference of which we have shown to be broadly invariant to cell death (see Supplementary Figure 8B), and comparable to Ref. 38. Hence, the number of mutations up

to generation 36 in the cell death model (as determined by Eq.(16)) is expected to be similarly distributed in the cell death model, prior to the cell death process occurring.

However, the death process may stochastically eliminate mutational foci. In Supplementary Figure 8C, we show that for generations $i^* \leq 32$, corresponding to mutational foci of size $\gtrsim 4$ (see Supplementary Figure 8D for the distribution of sizes for regions seeded at $i^* = 32$), the probability of extinction of such foci through cell death is negligible under this model of cell death ($< 2\%$ for $g \leq 7$). Hence, the amount of probability mass for X_{i^*} in Fig. 5C which may be affected by cell death is upper-bounded by 2% for generations $i^* \leq 32$. As shown in the inset of Supplementary Figure 8C, the probability of extinction decays extremely quickly as i^* decreases.

Mean size of pathological neuronal mutations (Fig. 5D)

We explore how the mean size of pathological neuronal mutations (Fig. 5D) is affected by this model of cell death, using the following model. If we assume that mutant regions in the brain are dilute enough such that no two mutated regions overlap (discussion below), we may write the total number of pathological cells (T_g) (Fig. 5E), for a given level of cell death, as

$$T_g = \sum_{i^*} \sum_{j=1}^{X_{i^*}} C_{i^*,g,j} \tag{26}$$

where $C_{i^*,g,j}$ is a random variable with the same distribution as $C_{i^*,g}$ (see Eq.(24)), with an additional index j to label each mutation event at generation i^*. X_{i^*} is the number of pathological mutations arising at generation i^*, see Eq.(16) where $N_b = 298$ is the number of pathological bases in the case genes. Eq.(26) sums all surviving cells from every mutation event j, over all generations i^*, for a given level of cell death g. The mean size of all surviving pathological regions (S_g) is therefore

$$S_g = \frac{T_g}{\sum_{i^*} \sum_{j=1}^{X_{i^*}} I(C_{i^*,g,j})} \tag{27}$$

where $I(x)$ is defined in Eq.(6). In Supplementary Figure 8E, we show that (using a typical value of $\lambda_m = 1.5 \times 10^{-9}$ mutations per base per division, which is comparable to the literature value from Tomasetti *et al.*[38] which is 0.64×10^{-9} mutations per base per division) the mean number of neurons per pathological region reduces with increasing cell death. Given non-zero cell death, the additional generations may result in mutant foci which are on average smaller than 1 neuron (due to Eq.(25), where $N_{\text{neu}} = 2^{36}$ and $i^* > 34$ when $g > 0$). Hence, single-neuron mutational foci are more common given cell death, which reduces the expected size of mutant foci, and so Fig. 5D is sensitive to this model of cell death.

Eqs.(26)-(27) rely upon the assumption that mutations are sufficiently dilute such that no two mutations overlap (otherwise cell death may remove a cell which is e.g. doubly mutated: this is not accounted for in Eq.(26)). However, we observe in Supplementary Figure 8F that, for the ranges of cell death explored here, the majority of simulated individuals have fewer than $\sim 10^6$ mutant neurons, corresponding to $\sim 0.0015\%$ of all neurons. Hence, our assumption of dilute mutant regions is reasonable.

Total number of pathological neurons (Fig. 5E)

Given the arguments in the previous section, we find that the distribution of the total number of pathologically mutated neurons per individual (see Fig. 5E) is significantly increased with non-zero cell death (see Supplementary Figure 8F, comparing $g = 7$ against $g = 0$ on a linear scale, $p < 10^{-12}$ one-sided Mann-Whitney U-test). This is also likely due to the additional rounds of growth introducing more single-cell mutations. However, the order of magnitude is largely unaffected ($\sim 10^6$).

Probability of 1 or more pathologically mutated regions at generation i^* (Fig. 5F)

We expect the probability of 1 or more pathologically mutated regions at generation i^*, for $i^* < 20$, (see Fig. 5F) remains unchanged, see the discussion relating to the mean size of pathological neuronal regions.

Number of regions regions per individual of 128 pathologically mutated cells (Fig. 5G)

We expect the number of regions per individual consisting of approximately 128 pathologically mutated cells (see Fig. 5G), to remain unchanged given non-zero cell death. Using the properties of the hypergeometric distribution (Eq.(24)), the probability of a region seeded at $i^* = 27$ to go extinct when $g = 1$ (a plausible value, see[40]) is 10^{-78}, hence we expect all such regions to remain in the adult brain under this model of cell death. The size of such regions is 112-144 neurons (95% confidence interval), hence the distribution is sufficiently constrained for 128 neurons to be indicative of the size of regions seeded at this generation.

To conclude, our central claim that somatic mosaicism is expected to exist in the adult human brain given our data and a crude model of neurodevelopment is expected to be robust when including this simple model of cell death.

Supplementary Note 2

Initial conditions for neurodevelopment

In our crude branching model of neurodevelopment, we assumed that neurodevelopment begins with a single founder cell. This is a clear simplification, since the central nervous system is derived from the ectoderm of the embryo, whereas the other germ layers give rise to other tissues and organs.[41] As a preliminary means of understanding the effect of neurodevelopment beginning from an initial founder pool of cells, we may modify our original model of neurodevelopment presented in the Main Text, such that neurodevelopment begins from a set of e.g. $2^{10} = 1024$ cells which are assumed to be without mutation; we refer to this modified model as the "progenitor pool model".

From Fig. 5F, we see that mutations occurring at generations $i^* \leq 10$ in the original model presented in the Main Text affect on the order of 1 in 1000 individuals, or fewer. The progenitor pool model differs from the original model only through enforcing that mutations in generations $i^* \leq 10$ occur with 0 probability (see Supplementary Figure 9A). Since mutations at $i^* \leq 10$ were rare in the original model, it is reasonable to assume that the distribution of inferred mutation rates for the progenitor pool model is highly similar to the original model (Fig. 5B). We therefore use the same distribution of mutation rates in Fig. 5B for the progenitor pool model (which are comparable to the mutation rate from Ref. 38), and consider the consequences of this alternative model through forward-simulation.

- Number of pathologically mutated regions (compare to Fig. 5C): Since the progenitor pool model is identical to the original model from generations $i^* = 11$ onwards, the analogue of Fig. 5C for the progenitor pool model is identical.
- Mean pathologically mutated region size (compare to Fig. 5D): The distribution for the mean pathologically mutated region size is almost unchanged under the progenitor pool model (Supplementary Figure 9B). This is because there are exponentially more mutation events as the generation number i^* increases, which dominates the mean region size in both models.
- Total number of pathological mutations (compare to Fig. 5E): The modal value for the total number of pathological mutations per individual shifts from 6.0×10^5 cells per individual in

the original model (Fig. 5E) to 5.2×10^5 cells per individual (Supplementary Figure 9C) in the progenitor pool model; however, these distributions show broad overlap.

- Probability of 1 or more mutations at generation i^* (compare to Fig. 5F): The analogue of Fig. 5E for the progenitor pool model is for all mutations at generations $i^* \leq 10$ to be set to $P(X_i \geq 1) = 0$. However, the observation of e.g. 11% of individuals possessing 3×10^5 spatially-contiguous pathologically mutated cells is robust under this simple modification of the original model, since this corresponds to $i^* = 16$ and therefore follows the same distribution as the original model.
- Number of pathological regions of size 128 (compare to Fig. 5G): This observation is unchanged since mutations occurring at $i^* = 27$ are independent of generations $i^* \leq 10$.

Overall, assuming that neurodevelopment begins with a population of ~ 1000 unmutated cells (or fewer) results in similar predictions (both quantitatively and qualitatively) to the original deterministic branching process for neurodevelopment. This is because, in the original model, if a mutation has not occurred by generation 10, the progenitor model is entirely equivalent to the original model. This can be seen in Supplementary Figure 9A, where a mutation which occurs at the point where the developing brain is the same size between the two models affects an identical amount of the final adult brain (i.e. the red and yellow shaded regions have the same area in both models). In the original model, $\sim 1/1000$ individuals or fewer experience a mutation within the first 10 divisions (Fig. 5F). So, for $\sim 99.9\%$ of individuals, the progenitor model is equivalent to the original model. These 99.9% are in such a vast majority that they wash out the statistical signal from the 0.1% who are differentially affected between the two models, as shown in Supplementary Figure 9B&C.

Supplementary Note 3

Asymmetric division in neurodevelopment

In our original model of neurodevelopment (see Main Text and Supplementary Methods), we assumed that every cell division resulted in two daughter cells which were both able to divide (symmetric division) until the final cell division. However, it is commonly thought that neurodevelopment proceeds in two phases: a proliferative phase mostly consisting of symmetric division, and a differentiative phase where each progenitor cell undergoes sequential rounds of asymmetric division.[42] In this case, we define asymmetric division as consisting of a progenitor cell dividing into another progenitor cell and a differentiated cell, where the differentiated cell ceases to divide.

We may modify the original model to include a simple model of asymmetric cell division, see Supplementary Figure 10A. In this asymmetric model of neurodevelopment, let I_s be the generation number of the final symmetric division, and I_a the total number of divisions which give rise to differentiated cells (asymmetric divisions plus the final division, where progenitor $\rightarrow$ differentiated + differentiated). Again assuming that we are modelling only the neuronal subpopulation, then the total number of neurons (N_{neu}) is

$$N_{\text{neu}} = 2^{I_s}(I_a + 1). \tag{28}$$

In Supplementary Figure 10B we see that asymmetric division induces a stochastic element to the number of daughters affected by a mutation occurring at generation i^*. Since asymmetric divisions affect the tips of the neurodevelopmental tree, they are consequently the most numerous events: it is therefore important that these possible stochastic paths are properly accounted for. There are three cases to consider for when a mutation occurs, and the corresponding number of mutated daughters (N_{i^*}) which the mutation gives rise to. Firstly,

$$N_{i^*} = 2^{I_s-(i^*+2)}(I_a + 1) \quad \text{if} \quad i^* \leq I_s - 2. \tag{29}$$

In this case, a mutation is guaranteed to be inherited by a progenitor cell on both strands of one of its chromosomes (see Supplementary Figure 7A), and affect all subsequent daughters. The second case corresponds to

$$N_{i^*} = \begin{cases} I_a & \text{w.p. } 1/2 \\ 1 & \text{w.p. } 1/2 \end{cases} \quad \text{if} \quad i^* = I_s - 1 \tag{30}$$

(where w.p. stands for "with probability"). Upon copying the parental DNA, the base at which a mutation arises initially affects only a single strand of DNA: this mutation must be copied into the subsequent generation for it to affect both strands of a single chromosome (see Supplementary Figure 7A). In the case denoted by Eq.(30), the single-stranded mutation is guaranteed to be inherited by a progenitor cell but, upon subsequent copying, the double-stranded mutation can either be inherited by a progenitor cell (affecting I_a subsequent daughters) or a single differentiated daughter cell. These two possibilities occur with equal probability. The third, and final, case is

$$N_{i^*} = \begin{cases} 0 & \text{w.p. } 1/2 \\ 1 & \text{w.p. } 1/4 \\ I_s + I_a - (i^* + 1) & \text{w.p. } 1/4 \end{cases} \quad \text{if} \quad i^* \geq I_s. \tag{31}$$

In this case, a single-stranded mutation may be inherited by a differentiated cell w.p. 1/2, meaning that the single-stranded is never subsequently copied (as it resides in a non-dividing cell). We do not count mutations which only appear on a single strand of DNA for simplicity, hence the number of mutated cells which arise from this event is 0, w.p. 1/2. However, if the single-stranded mutation is inherited by a progenitor, upon subsequent copying, the double-stranded mutation may either be inherited by another progenitor or by a differentiated cell (each with probability 1/2). If the double-stranded mutation is inherited by a differentiated cell, then we count 1 mutant. Otherwise, the mutation will affect all subsequent progenitors and differentiated cells from that lineage, affecting $(I_s + I_a - (i^* + 1))$ daughters in total. Since these final two possibilities are conditioned upon a single-stranded mutation being inherited by a progenitor (w.p. 1/2), the overall probability of these two possibilities is 1/4. Note that the three outcomes described above are therefore multinomially distributed.

In the monkey foetus, asymmetric cell division lasts for 30-60 days.[43] Based on this, we use a liberal estimate of 12 weeks for the duration of asymmetric division, and a cell division time of 24 hours, yielding $I_a = 84$ asymmetric divisions. Letting $N_{\text{neu}} = 86.1 \times 10^9$ [35] and rearranging Eq.(28) for I_s yields $I_s = 30$ (corresponding to a 6% overestimate in the number of neurons according to[35]).

We use the literature value of $\lambda_m = 6.4 \times 10^{-10}$ per base per cell division[38] as the mutation rate in the discussion below for where we have performed forward-simulation (10^4 simulated individuals). The mutation rate used from Tomasetti *et al.*[38] is broadly compatible with the distribution found in Fig. 5B. We now investigate the implications of the asymmetric model of neurodevelopment relative to the original model presented above.

- Number of pathologically mutated regions (compare to Fig. 5C): In Supplementary Figure 10C, we find that the number of pathologically mutated regions increases exponentially up to generation I_s. This is in agreement with the original model (Fig. 5C), since the models are identical in structure up to generation I_s. The variance is smaller since we have used a single value for the mutation rate, rather than a distribution. After generation I_s, the number of pathological mutations at each subsequent generation is identically distributed (as shown by the plateau for $i^* \geq I_s$) because each generation number has an identical number of cells associated with it.

- Mean pathologically mutated region size (compare to Fig. 5D): The modal value for the mean number of neurons per pathological region is 31 neurons, as opposed to 8 neurons in the original

model (Fig. 5D). This is expected because under the original model there are exponentially more cells in later generations, which give rise to smaller mutational foci. However, in the asymmetric model, there are the same number of cells per generation during the differentiative phase, and therefore small foci are not as heavily represented in the asymmetric model.

- Total number of pathological mutations (compare to Fig. 5E): In Supplementary Figure 10E, we see that the most probable number of pathologically mutated neurons per individual is 1.6×10^6, which is 2.7 times greater than the original model (6×10^5, Fig. 5E); this can be accounted for, in part, by the observation that the asymmetric model has a value of N_{neu} which is 30% larger than the original model. We note that the predictions for the total number of mutations in both models are comparable in order of magnitude.

- Probability of 1 or more mutations at generation i^* (compare to Fig. 5F): Since the asymmetric model is identical to the original model up to generation $I_s = 30$, Fig. 5F also holds for the asymmetric model, albeit with different numbers of mutated cells corresponding to each value of i^*. Substituting into Eq.(29), 79% of individuals are expected to possess at least 1 region consisting of 2.2×10^4 spatially contiguous pathologically mutated cells, 11% of individuals possess a region of 3.5×10^5 cells, and 1% of individuals possess a region of 2.8×10^6 cells. These values are all comparable in magnitude to those displayed in Fig. 5F.

- Number of pathological regions of size 128 (compare to Fig. 5G): Since generation $i^* = 27 < I_s - 2$, the distribution for the number of regions seeded at generation $i^* = 27$ is identical to the original model (given the same set of mutation rates, see Fig. 5G). Note that, under the asymmetric model, mutations seeded at generation $i^* = 27$ correspond to mutant regions consisting of 170 cells.

Hence, under a simplified asymmetric model of neurodevelopment, the total number of pathological mutations is approximately doubled and the mean mutated region size is approximately tripled. Overall, this simple asymmetric model of neurodevelopment predicts a prevalence of somatic mosaicism which is similar in order of magnitude to the original model presented in the Main Text.

Supplementary Note 4

Intuition for model robustness

In Supplementary Note 1, Supplementary Note 2 and Supplementary Note 3, we found that many of our quantitative and qualitative observations regarding somatic mosaicism during neurodevelopment were robust to simple alterations in the model structure, which addressed issues of cell death, progenitor founder effects and asymmetric division. Here, we provide an order-of-magnitude calculation for why this is expected to be the case.

Suppose at some instance in time (t) during neurodevelopment there exist $N(t)$ cells. Suppose $N(t)$ is large enough such that, upon replication of each of the $N(t)$ cells once, 1 pathological mutation occurs on average. This scenario may still be modelled with a simple binomial distribution. We may therefore apply Eq.(16), where we substitute 2^{i^*} with $N(t)$ and set $\mathbb{E}(X_{i^*}) = 1$ (where X_{i^*} is a random variable denoting the number of mutations which arise in copying $N(t)$ cells). This yields

$$1 = 4N_b N(t)\lambda_m. \tag{32}$$

Substituting $\lambda_m = 6.4 \times 10^{-10}$ mutations per base per doubling[38] and $N_b = 298$ bases (mutations in which are pathological) yields $N(t) = 1.3 \times 10^6$ cells. We neglect subtleties relating to lags associated with copying each strand of DNA (see Eq.(4) and Supplementary Figure 7A). We now assume the following

1. We require *unbiased* replication of all daughters *after* the mutation event when the brain consisted of $N(t)$ cells (unbiased meaning that each cell gives rise to the same number of daughter cells).

2. We require daughters to remain spatially proximal during this replication.

Given assumption (1), we expect the fraction $\sim 1/N(t)$ of all subsequent cells to carry this mutation. (Note that accounting for the copying lag will reduce this to $\sim 1/4N(t)$, but is on the same order of magnitude.) Using $N_{\rm neu} = 86.1 \times 10^9$ neurons results in the prediction that every individual harbours, on average, 1 pathologically mutated region consisting of approximately $N_{\rm mut}$ spatially-contiguous cells, where

$$N_{\rm mut} = N_{\rm neu}/N(t) = 6.6 \times 10^4 \text{ cells}, \tag{33}$$

and spatial contiguity follows from assumption (2). Increasing the value on the left hand side of Eq.(32) (i.e. the number of mutation events at a particular stage of neurodevelopment) increases $N(t)$ and reduces $N_{\rm mut}$ according to Eq.(33). This is the qualitative essence of somatic mosaicism: mutations which are more common are smaller, and vice versa.

No details relating to the process which generated the $N(t)$ cells were assumed, so founder effects are unimportant prior to when the cell consisted of $N(t)$ cells. All of the models explored above satisfy these two assumptions, so this order-of-magnitude calculation is expected to approximately hold.

We may check this argument by examining Fig 5F at $i^* = 20$, since all of the above models satisfy Fig 5F at $i^* = 20$. This states that 80% of individuals are expected to carry mutations of size 1.6×10^4 cells. This observation is consonant with the above argument to within an order of magnitude. We therefore expect models of neurodevelopment which satisfy the above 2 properties to show somatic mosaicism where it is common for individuals to have mutant regions of $\sim 10^4$ cells, given a mutation rate between 10^{-10}–10^{-9} mutations per base per division. This argument also includes combinations of the above model perturbations, e.g. the progenitor pool model + asymmetric division. A notable example of where this argument does not apply is in the case of large cellular migration, which we discuss in Supplementary Note 5.

We note that a natural generalization of our inference approach would be to relax the assumption of spatial contiguity of the sampled cells, allowing for the sample size to correspond to the full 1 cm^3 block of tissue. However, it is not obvious that altering the sample size will have any effect upon the inferred mutation rate. Defining $N_{\rm br}$ as the total number of cells in the brain, Eq.(4) and Eq.(16) imply that the average total number of mutated cells in the adult brain which originate from a particular generation number (N_{i^*}) is $N_{i^*} = N_b \lambda_m N_{\rm br}$, where N_{i^*} is itself independent of the generation number i^*. Furthermore, consider dividing a brain into 'voxels' each of the same volume as the sample size. The total number of detectably mutated cells across all voxels (N_d) equals the number of detectably mutated cells per mutated voxel (which is proportional to the variant allele fraction, VAF), multiplied by the total number of voxels which contain a detectable mutation. This is true for any voxel size, and independent of the voxel size itself. Given that a threshold sensitivity for detecting mutations implies that detectable mutations tend to originate from a narrow band of i^* values, simply determining the proportion of mutated voxels, the VAF, and equating $N_d = N_{i^*}$, allows λ_m to be inferred. Such a calculation is independent of the voxel (or sample) size. Thus, altering the sample size will not obviously affect the inferred λ_m. We further note that all of our results are qualitatively the same if we had simply used the mutation rate from Ref. 38.

Supplementary Note 5

Cell migration and spatial correlations in neurodevelopment

In the crude branching model of neurodevelopment, we assumed that cells which were in close spatial proximity shared a similar developmental lineage. However, cells have the ability to migrate during development.[44] If cells have appreciable migration during development, then this reduces spatial correlations in the mutational profile of brain cells.

The extent of cell migration informs upon the degree to which our original model is perturbed from true neurodevelopment. It has been suggested that neurons are established by radial glial cells, forming functional neocortical columns.[45] These columns are between 200–1000 μm across.[46] If most migration were to occur on the micron scale, our experimental approach would be largely insensitive to this, since each sample was approximately 1 cm^3 in size and homogenized. However, tangential migration has also been observed,[44] where cells move across different subdivisions of the forebrain.

In the following section, we provide an alternative perspective to neurodevelopment where the brain consists of independent regions of some characteristic size. Such a model better represents the diffusive aspects of neurodevelopment, although makes other assumptions which are arguably less plausible than the original model presented in the Main Text. We note that we find the total number of pathological mutations in all of the models investigated here to be on the order of 10^5-10^6 cells (see Fig. 5E, Supplementary Figure 8F, Supplementary Figure 9C, Supplementary Figure 10E and Supplementary Figure 11E).

Supplementary Note 6

Constant region size model

Summary

In this section, we consider an alternative model where the brain is modelled as consisting of independent regions of some characteristic size f, which are homogeneously mutated at the mean VAF experimentally observed in the case genes in the case patients. $f = 1$ suggests that if the brain is mutated at a particular base pair then this mutation is present across the entire brain. However, if $f < 1$ then there exists $1/f$ regions in the brain, each consisting of Nf cells, where N is the number of cells in the brain. We assume that each region has a probability p_m of being homogeneously mutated at a particular base pair in the case genes (Supplementary Figure 11A). This model does not make a commitment to a tree-like development instead assuming that mutations occupy characteristic volumes of tissue. This makes the model, in some sense, less realistic but it has the virtue of being a different perspective that allows us to probe the robustness of our observations. This model also lends itself more naturally to the diffusive nature of neurodevelopment (when $f < 1$), unlike the crude branching model above.

The parameters f and p_m were considered as shared amongst all patients, for parsimony. We investigated the effect of varying f by orders of magnitude through approximate Bayesian computation (see below). We again restricted our attention to patients with neurodegenerative disease to avoid sampling bias in our extrapolations. We found that, under this model, the relative size of independent regions was not strongly constrained by the data (Supplementary Figure 11C), finding only that large region sizes $\gtrsim 10\%$ are unlikely. (The tree-like structure considered in the Main Text model puts a much stronger constraint on the distribution of sizes of patches of mutated cells.)

The data did constrain the mean number of mutated regions per individual such that the lower bound was approximately 1 region per individual ($0.942 \pm 6.6 \times 10^{-3}$ mutated regions per individual, 5th percentile of posterior distribution, see Supplementary Figure 11D). Consequently, we found

that 63% of simulated individuals carried non-zero levels of pathologically mutated cells in case genes, carrying between 0.33–5.0 $\times 10^5$ pathologically mutated cells per affected individual (95% Bayesian credible interval, see Supplementary Figure 11E). This number of cells with pathological mutations is in broad agreement with Fig. 5E. Thus, even this remarkably crude model, that makes no commitments to a developmental hierarchy, suggests that zones of pathological mutations occur in the brains of most people. That we observe a subset of simulated individuals lacking mutated regions (and therefore pathologically mutated cells) is due to our broad priors in f and p_m allowing for independent brain regions to be large enough that it is unlikely for any pathological regions to arise in an individual (this limit is $f \gtrsim \tilde{p}p_m$ where $\tilde{p} = 298/132617$ is the probability of a region being pathologically mutated, given that it is mutated, see below – Supplementary Figure 11G).

Approximate Bayesian computation for fixed region size model

We consider the brain as consisting of 'regions', each having a relative mass (f) where $0 < f \leq 1$, implying that each brain contains $1/f$ such regions. For the subset of the data we considered (the case genes in the case cohort), all samples measuring positive for somatic mutations corresponded to unique genes in unique patients. Consequently, we used the parsimonious model that positive samples were homogeneously mutated in a particular case gene at the average VAF (v) determined experimentally ($v = 0.87\%$). We modelled each region as having an independent probability p_m of being homogeneously mutated at a particular base of the case genes.

Defining Y_i as the number of mutated regions in the brain of a single individual (i), then Y_i follows a binomial distribution

$$Y_i \sim \text{Binom}\left(\frac{1}{f}, p_m\right) \tag{34}$$

where Binom(n, p) is a binomial trial with n attempts each with probability p of success. Note that $1/f$ is constrained to be an integer, and may be obtained by rounding $1/f$ for a given f. We then model the measurement process for each individual as consisting of n_i samples. Since there are Y_i patches, each of which occupies a fraction f of the brain, then fY_i gives the total fraction of the brain which is mutated, where $0 \leq fY_i \leq 1$. This results in M_i samples positive for mutation for individual i, with

$$M_i \sim \text{Binom}(n_i, fY_i). \tag{35}$$

Note that we have modelled all individuals as sharing the only two free parameters in the model: f and p_m.

To infer these parameters, we used an approximate Bayesian computation (ABC) rejection algorithm. We used the uninformative priors

$$\log(f) \sim \text{unif}\left(\log\left(\frac{3415}{86 \times 10^9}\right), 0\right) \tag{36}$$

$$p_m \sim \text{unif}(0, 1) \tag{37}$$

where we chose to use a prior for f which was uniform over log-space, since we were uncertain of its value over orders of magnitude. The lower limit of the prior for f was chosen such that f could not be smaller than the approximate relative size occupied by a single sample i.e. 3415 cells out of the total number of cells in the brain. We have idealized the brain as consisting purely of neurons, of which there are approximately 86×10^9 per brain.[35]

We simulated each patient by drawing (f, p_m) from the prior, and determining M_i with Eq.(35). We then used the following summary statistics for the simulated model ($\mathbf{S}$)

$$\mathbf{S} = \left(\sum_{i=1}^{40} M_i, \sum_{i=1}^{40} I(M_i)\right) \tag{38}$$

where I is the indicator function defined in Eq.(6), and the upper summation limit corresponds to the number of case patients. The first element counts the total number of positive samples across all patients, whereas the second element counts the total number of affected patients. The summary statistic for the data is $\hat{\mathbf{S}} = (6, 6)$. We used the distance metric

$$\rho(\mathbf{S}, \hat{\mathbf{S}}) = \sum |\mathbf{S} - \hat{\mathbf{S}}| \leq \epsilon \tag{39}$$

and required the tolerance $\epsilon = 0$ to accept $(f,\ p_m)$ as a draw from the approximate posterior $P(f, p_m|\rho(\mathbf{S}, \hat{\mathbf{S}}(\mathcal{D})) = 0) \approx P(f, p_m|\mathcal{D})$ for data $\mathcal{D}$. This therefore enforces the ABC rejection algorithm to only accept samples where every mutation occurs once in each affected patient (as observed in the data), and that the correct number of mutant samples and patients are observed overall.

Given posterior samples of (f, p_m), we may also infer the distribution of the number of pathologically mutated neurons per brain. Dropping the patient subscript i, and using Eq.(34), the number of pathologically mutated regions per patient $(\tilde{Y})$ is

$$\tilde{Y} \sim \text{Binom}(Y, \tilde{p}) \tag{40}$$

where $\tilde{p}$ is the probability of a mutated region being pathological, which we take as 298/132617, being the proportion of base pairs in the case genes being associated with pathology. From this, we approximate the number of pathologically mutated cells (N_p) as

$$N_p \approx \tilde{Y} \cdot (Nf) \cdot v \tag{41}$$

where N = number of neurons per brain and v = is the proportion of mutant cells in a positive sample, which we take as the average VAF $v = 0.87 \times 10^{-2}$. The term (Nf) is the number of neurons per region.

It is instructive to observe that the mean number of mutant cells, $\langle N_p \rangle$, for fixed f and p_m is $\langle N_p \rangle = Nfv\langle \tilde{Y} \rangle = Nfv\tilde{p}\langle Y \rangle$. Using Eq.(34), $\langle N_p \rangle = Nfv\tilde{p}p_m/f = Nv\tilde{p}p_m$ *which is independent of* f. Note that if $f \ll \tilde{p}p_m$ then $\langle \tilde{Y} \rangle \gg 1$, in which case an individual is likely to harbour at least one pathological mutation (see Supplementary Figure 11G). Substituting $N = 86 \times 10^9$ neurons, $v = 0.87 \times 10^{-2}$, and the maximum *a posteriori* estimate of $(p_m)_{MAP} = 6/119$, yields $\langle N_p \rangle = 1.70 \times 10^5$ pathologically mutated neurons per brain, which approximately corresponds to the peak shown in Supplementary Figure 11E.

Supplementary References

1. Stein H*, et al.* CD30(+) anaplastic large cell lymphoma: a review of its histopathologic, genetic, and clinical features. *Blood* **96**, 3681-3695 (2000).

2. Aukema SM*, et al.* Double-hit B-cell lymphomas. *Blood* **117**, 2319-2331 (2011).

3. Scheicher R*, et al.* CDK6 as a key regulator of hematopoietic and leukemic stem cell activation. *Blood* **125**, 90-101 (2015).

4. Kim M*, et al.* Quantitative comparison of CDKN2B methylation in pediatric and adult myelodysplastic syndromes. *Acta Haematol* **130**, 115-121 (2013).

5. Genovese G*, et al.* Clonal hematopoiesis and blood-cancer risk inferred from blood DNA sequence. *N Engl J Med* **371**, 2477-2487 (2014).

6. Martens JH. Acute myeloid leukemia: a central role for the ETS factor ERG. *Int J Biochem Cell Biol* **43**, 1413-1416 (2011).

7. Zhang MY*, et al.* Germline ETV6 mutations in familial thrombocytopenia and hematologic malignancy. *Nature genetics* **47**, 180-185 (2015).

8. Nikoloski G*, et al.* Somatic mutations of the histone methyltransferase gene EZH2 in myelodysplastic syndromes. *Nature genetics* **42**, 665-667 (2010).

9. Baxter EJ*, et al.* Acquired mutation of the tyrosine kinase JAK2 in human myeloproliferative disorders. *Lancet* **365**, 1054-1061 (2005).

10. Macchi P*, et al.* Mutations of Jak-3 gene in patients with autosomal severe combined immune deficiency (SCID). *Nature* **377**, 65-68 (1995).

11. Ballmaier M*, et al.* c-mpl mutations are the cause of congenital amegakaryocytic thrombocytopenia. *Blood* **97**, 139-146 (2001).

12. Bhatia K, Huppi K, Spangler G, Siwarski D, Iyer R, Magrath I. Point mutations in the c-Myc transactivation domain are common in Burkitt's lymphoma and mouse plasmacytomas. *Nature genetics* **5**, 56-61 (1993).

13. Borrow J, Goddard AD, Sheer D, Solomon E. Molecular analysis of acute promyelocytic leukemia breakpoint cluster region on chromosome 17. *Science* **249**, 1577-1580 (1990).

14. Wong TN*, et al.* Role of TP53 mutations in the origin and evolution of therapy-related acute myeloid leukaemia. *Nature* **518**, 552-555 (2015).

15. Cimino G*, et al.* Cloning of ALL-1, the locus involved in leukemias with the t(4;11)(q21;q23), t(9;11)(p22;q23), and t(11;19)(q23;p13) chromosome translocations. *Cancer Res* **51**, 6712-6714 (1991).

16. Smith ML, Cavenagh JD, Lister TA, Fitzgibbon J. Mutation of CEBPA in familial acute myeloid leukemia. *The New England journal of medicine* **351**, 2403-2407 (2004).

17. Michaud J*, et al.* In vitro analyses of known and novel RUNX1/AML1 mutations in dominant familial platelet disorder with predisposition to acute myelogenous leukemia: implications for mechanisms of pathogenesis. *Blood* **99**, 1364-1372 (2002).

18. Braak H, Braak E. Neuropathological stageing of Alzheimer-related changes. *Acta neuropathologica* **82**, 239-259 (1991).

19. Thal DR, Rub U, Orantes M, Braak H. Phases of A beta-deposition in the human brain and its relevance for the development of AD. *Neurology* **58**, 1791-1800 (2002).

20. Mirra SS*, et al.* The Consortium to Establish a Registry for Alzheimer's Disease (CERAD). Part II. Standardization of the neuropathologic assessment of Alzheimer's disease. *Neurology* **41**, 479-486 (1991).

21. Braak H, Del Tredici K, Rub U, de Vos RA, Jansen Steur EN, Braak E. Staging of brain pathology related to sporadic Parkinson's disease. *Neurobiology of aging* **24**, 197-211 (2003).

22. McKeith IG. Consensus guidelines for the clinical and pathologic diagnosis of dementia with Lewy bodies (DLB): report of the Consortium on DLB International Workshop. *Journal of Alzheimer's disease : JAD* **9**, 417-423 (2006).

23. Kumar P, Henikoff S, Ng PC. Predicting the effects of coding non-synonymous variants on protein function using the SIFT algorithm. *Nat Protoc* **4**, 1073-1081 (2009).

24. Chun S, Fay JC. Identification of deleterious mutations within three human genomes. *Genome Res* **19**, 1553-1561 (2009).

25. Li B*, et al.* Automated inference of molecular mechanisms of disease from amino acid substitutions. *Bioinformatics* **25**, 2744-2750 (2009).

26. Kircher M, Witten DM, Jain P, O'Roak BJ, Cooper GM, Shendure J. A general framework for estimating the relative pathogenicity of human genetic variants. *Nature genetics* **46**, 310-315 (2014).

27. Alexandrov LB*, et al.* Signatures of mutational processes in human cancer. *Nature* **500**, 415-421 (2013).

28. Li H, Durbin R. Fast and accurate short read alignment with Burrows-Wheeler transform. *Bioinformatics* **25**, 1754-1760 (2009).

29. Li H*, et al.* The Sequence Alignment/Map format and SAMtools. *Bioinformatics* **25**, 2078-2079 (2009).

30. Purcell S*, et al.* PLINK: a tool set for whole-genome association and population-based linkage analyses. *Am J Hum Genet* **81**, 559-575 (2007).

31. DePristo MA*, et al.* A framework for variation discovery and genotyping using next-generation DNA sequencing data. *Nat Genet* **43**, 491-498 (2011).

32. McKenna A*, et al.* The Genome Analysis Toolkit: a MapReduce framework for analyzing next-generation DNA sequencing data. *Genome Res* **20**, 1297-1303 (2010).

33. Van der Auwera GA*, et al.* From FastQ data to high confidence variant calls: the Genome Analysis Toolkit best practices pipeline. *Curr Protoc Bioinformatics* **43**, 11 10 11-33 (2013).

34. Quinlan AR, Hall IM. BEDTools: a flexible suite of utilities for comparing genomic features. *Bioinformatics* **26**, 841-842 (2010).

35. Azevedo, F. A. *et al.* Equal numbers of neuronal and nonneuronal cells make the human brain an isometrically scaled-up primate brain. *J. Comp. Neurol.* **513**, 532-541 (2009).

36. Roach, J. C. *et al.* Analysis of genetic inheritance in a family quartet by whole-genome sequencing. *Science* **328**, 636-639 (2010).

37. Ross, S. M. *et al. Stochastic processes*, vol. 2 (John Wiley & Sons New York, 1996).

38. Tomasetti, C., Vogelstein, B. & Parmigiani, G. Half or more of the somatic mutations in cancers of self-renewing tissues originate prior to tumor initiation. *Proc. Natl. Acad. Sci. U.S.A.* **110**, 1999-2004 (2013).

39. Yamaguchi, Y. & Miura, M. Programmed cell death in neurodevelopment. *Dev. Cell* **32**, 478-490 (2015).

40. Oppenheim, R. W. Cell death during development of the nervous system. *Annu. Rev. Neurosci.* **14**, 453-501 (1991).

41. Vogel, F. & Motulsky, A. G. *Vogel and Motulsky's Human Genetics: Problems and Approaches* (Springer Science & Business Media, 2013).

42. Florio, M. & Huttner, W. B. Neural progenitors, neurogenesis and the evolution of the neocortex. *Development* **141**, 2182-2194 (2014).

43. Rakic, P. A small step for the cell, a giant leap for mankind: a hypothesis of neocortical expansion during evolution. *Trends Neurosci.* **18**, 383-388 (1995).

44. Marin, O. & Rubenstein, J. L. Cell migration in the forebrain. *Annu. Rev. Neurosci.* **26**, 441-483 (2003).

45. Noctor, S. C., Flint, A. C., Weissman, T. A., Dammerman, R. S. & Kriegstein, A. R. Neurons derived from radial glial cells establish radial units in neocortex. *Nature* **409**, 714-720 (2001).

46. Horton, J. C. & Adams, D. L. The cortical column: a structure without a function. *Philos. Trans. R. Soc. Lond. B Biol. Sci.* **360**, 837-862 (2005).
