# Supplementary material for: High prevalence of focal and multi-focal somatic genetic variants in the human brain
Source: Nat Commun. 2018 Oct 15;9:4257. doi: 10.1038/s41467-018-06331-w (PMC6189186; doi:10.1038/s41467-018-06331-w)
Supplement: Supplementary file 3 — Description of Additional Supplementary Files [file 41467_2018_6331_MOESM3_ESM.pdf]

Supplementary Data 1. Annotation of the 39 variants detected. The mutation number, together with individual case number (Supplementary Table 1), and brain region (or blood) are shown together with the amino-acid change are shown. In addition, the Variant Allele Frequency (VAF), together with the corrected p-vale for this variant compared against all other samples from other individuals and other samples in the same individual are shown. Finally, whether the same variant was previously detected by Jaiswal et al[1], and the final variant classification (SRM=Single Regional Mutation, NP = Not Present, MRM = Multiple Region Variant) are shown.
